# Study of the Chemical Composition of *Rosa beggeriana* Schrenk’s Fruits and Leaves

**DOI:** 10.3390/plants12183297

**Published:** 2023-09-18

**Authors:** Aigerim Aituarova, Galiya E. Zhusupova, Aizhan Zhussupova, Samir A. Ross

**Affiliations:** 1 Department of Chemistry and Technology of Organic Substances, Natural Compounds and Polymers, NPJSC Al-Farabi Kazakh National University, Al-Farabi Ave. 71, Almaty 050040, Kazakhstan; zhusupova@gmail.com; 2 Department of Molecular Biology and Genetics, NPJSC Al-Farabi Kazakh National University, Al-Farabi, Ave. 71, Almaty 050040, Kazakhstan; aizhan.zhusupova@gmail.com; 3 School of Pharmacy, University of Mississippi, P.O. Box 1848, Oxford, MS 38677, USA; sross@olemiss.edu; 4 School of Pharmacy, S.D. Asfendiyarov Kazakh National Medical University, Almaty 050000, Kazakhstan

**Keywords:** *Rosa beggeriana* Schrenk, 3β,23-dihydroxyurs-12-ene, β-sitosterol, betulin, (+)-catechin, lupeol, ethyl linoleate, ethyl linolenoate

## Abstract

*Rosa* species are widely used in folk medicine in different countries of Asia and Europe, but not all species are studied in-depth. For instance, *Rosa beggeriana* Schrenk, a plant which grows in Central Asia, Iran, and some parts of China, is little described in articles. Column and thin-layer chromatography methods were used to isolate biologically active substances. From a study of fruits and leaves of *Rosa beggeriana* Schrenk, a large number of compounds were identified, seven of which were isolated: 3β,23-dihydroxyurs-12-ene (**1**), β-sitosterol (**2**), betulin (**3**), (+)-catechin (**4**), lupeol (**5**), ethyl linoleate (**6**), and ethyl linolenoate (**7**). Their structures were elucidated by ^1^H, DEPT and ^13^C NMR spectroscopy, mass spectrometry, and GC-MS (gas chromatography–mass spectrometry). The study also identified the structures of organic compounds, including volatile esters and acids. Consequently, comprehensive data were acquired concerning the chemical constitution of said botanical specimen.

## 1. Introduction

The genus *Rosa* holds big commercial significance and is renowned in the domain of folk medicine. Numerous wild species within this genus have played a pivotal role in the development of valuable and economically viable cultivars of ornamental roses [1]. Despite the relative under-examination of certain rose species, several of them possess significant potential due to the presence of rosehip fruits. Commercially traded rosehip fruit is derived from several different species. They are long-lived woody perennials found mainly on forest margins and in disturbed habitats, such as roadsides and open fields. The genus *Rosa* (Rosaceae) has around 150–200 species [1,2,3]. Roses have also been cultivated since ancient times as medicinal plants in many countries across Europe and Asia. Rosehips contain many pharmacologically active compounds, such as organic acids, vitamin C and E, flavonoids, carotenoids, and tannins. Therapeutic properties and benefits of rosehips are their nourishing, mild laxative, mild diuretic, mild astringent, diuretic, ophthalmic and tonic effects [1,4,5,6]. *Rosa* extracts derived from these plants are also widely used in cosmetics, promising antioxidant and moisturizing effects [1,5,6,7,8]. All parts of this wild rose have been used in Asian folk medicine [4,5,9,10,11].

The intrinsic value of rosehip fruit has been acknowledged for centuries; however, efforts have only recently been made to domesticate and cultivate wild roses specifically for their fruit and to advance agronomic techniques in this regard. This shift in focus can be attributed to an enhanced comprehension of the pivotal role that dietary fruits play in enhancing human health and mitigating disease risks [12]. The Rosaceae family is one of the most employed as a consolidated source of phytoproducts with functional properties [13]. Within this family, the genus *Rosa* provides various species, and their essential oils possess a wide range of applications as flavor, fragrance, and additive in cosmetic and toiletries [14]. In addition to their aromatic composition, many *Rosa* species from all over the word have been evaluated for their food-related biological properties and multiple functional uses have been suggested [15,16,17,18,19]. For example, teas made from the fruits of *Rosa canina* have mild laxative and diuretic tendencies [12]. Rosehips have a longstanding history of utilization in folk medicine spanning centuries, primarily for the prevention and treatment of various ailments such as the common cold, influenza-like infections, fever, infectious diseases, vitamin C deficiency, general exhaustion, gastritis and gastric ulcers prevention, diarrhea, gallstones and gallbladder discomforts, urinary tract diseases and discomforts, as well as for their potential anti-inflammatory, anti-obesity, anticancer, and diabetes management properties. Furthermore, rosehips have been employed to address inadequate peripheral circulation concerns [5,6,15,16,17,18,20,21]. Mixed with small amount of vinegar, rosehips were used as an antidote for the treatment of iron toxicity [20,22]. For nutritional purposes, fruits are used for the production of different products like tea, marmalade, jam, stewed fruit, wine, and juices [23]. Ground in a hand mill and cooked with milk, they could be used as children’s snack and baby food as reported by the latter authors. The functional properties of some *Rosa* species are attributable to a wide range of bioactive ingredients, such as minerals, flavonoids, tannins, anthocyanin, organic acids, phenolic compounds fatty acids, volatile oils, ascorbic acid, phenols, and sugar. Through the examination of a species within the same taxonomic family as the subject of investigation, co-occurring within the identical geographical region, it becomes feasible to assess the potential anticancer properties of compounds derived from Begger’s rosehip [5,24,25,26,27,28].

*Rosa beggeriana* Schrenk is an indigenous species predominantly distributed in Central Asia (Kazakhstan, Kyrgyzstan), China (Xinjiang Uygur Autonomous Region), and Iran [29,30,31,32,33]. It has been identified as an essential resource for hybridization purposes, particularly in the development of cold-resistant germplasm, when combined with contemporary rose varieties [29]. Begger’s rosehip contains a large number of compounds with antioxidant activity, including activity against cancer cells. Rosehip hips have a high concentration of the carotenoid lycopene, which is considered a compound with a powerful antioxidant effect and is used as a therapeutic and prophylactic agent for various diseases, including cancer. Begger’s rosehip extracts display cytotoxicity and antiproliferative properties against human liver and breast cancer cells, which might be associated with the presence of polyphenols in it [34].

According to the studies mentioned earlier, we can tell that Begger’s rosehips have not been studied sufficiently even though other species are very well known in the folk medicine of different countries.

## 2. Results

### 2.1. Identification of the Isolated Compounds from Leaves of Rosa beggeriana Schrenk

Utilizing column chromatography, a total of seven distinct substances were successfully isolated from both the fruits and leaves of the wild rose species known as Begger (Rosa sp.). The isolated substances encompassed a diverse range of chemical classes, including triterpenoids, catechins, and fatty acid esters. Remarkably, 3β,23-dihydroxyurs-12-ene had not been previously reported in this particular plant species. Additionally, no NMR characterization data were available for this newly isolated substance [35,36,37]. Apart from this novel isolate, the triterpenoids, catechins, and a mixture of fatty acid esters were also successfully identified and isolated from the aforementioned plant material.

### 2.2. GC-MS Data

GC-MS analysis was used to obtain data on the fatty acid composition of the leaves (Table 1) and fruits (Table 2) of *Rosa beggeriana* Schrenk.

When comparing the two tables, a richer composition of the fatty acids in fruits can be observed (Table 1 and Table 2).

### 2.3. NMR Data

#### 2.3.1. Identification of the Isolated Compounds from Leaves of *Rosa beggeriana* Schrenk

**Figure 1:**
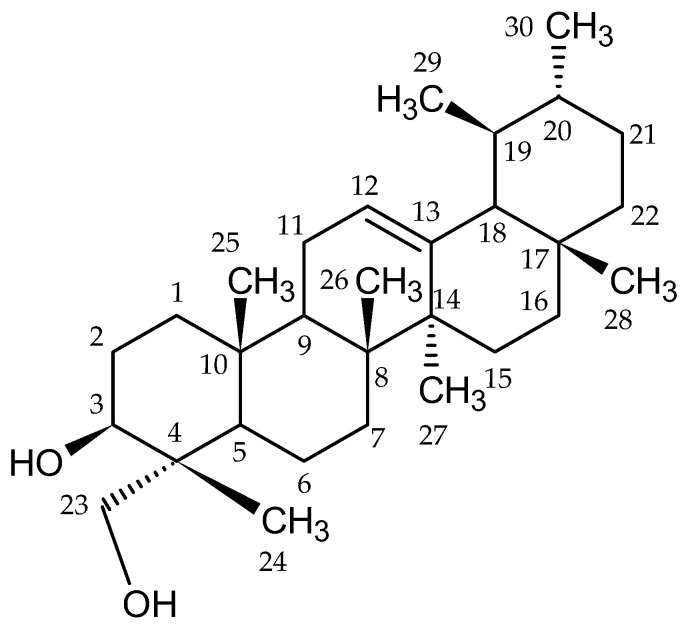
Chemical structure of 3β,23-dihydroxyurs-12-ene (**1**).

From the ethanol extract (45 g) of *Rosa beggeriana* Schrenk (553 g), 3β,23-dihydroxyurs-12-ene (**1**) [35,36,37] (21 mg) was isolated. The chemical structures are shown in Figure 1.

NMR data for Compound **1** were not found in literature. Hence, the analysis of the NMR spectra (Figures S1–S4) and the comparison of the spectroscopic data (Table 3) with those compounds that have a similar structure and described in the literature [35,36,37] allowed the identification of the compound **1**. The mass spectra also allowed us to identify compound **1**. Also, the melting point for compound **1** was 226–229 °C.

**Figure 2:**
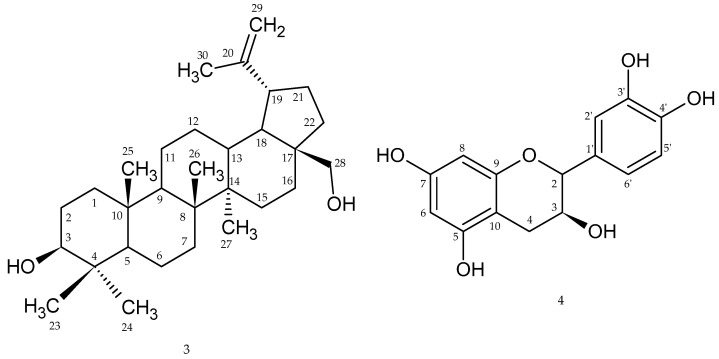
Chemical structure of betulin (**3**) and (+)-catechin (**4**).

From the ethanol extract (45 g) of *Rosa beggeriana* Schrenk (553 g), betulin (**3**) [38,39] (42.5 mg) and (+)-catechin (**4**) [40,41,42] (40.0 mg) were isolated. The chemical structures are shown in Figure 2.

Compound **3** (betulin) was identified by its characteristic ^1^H NMR (400 MHz; CDCl_3_) δ: 1.57, 1.27 (2H, s, H-1), 1.70 (2H, s, H-2), 3.21 (2H, dd, H-2), 0.96 (1H, s, H-5), 1.38 (2H, s, H-6), 1.57, 1.38 (2H, s, H-7), 1.38 (2H, s, H-11), 1.38 (2H, s, H-12), 1.57, 1.27 (2H, s, H-15), 1.57, 1.27 (2H, s, H-16), 1.92 (1H, m, H-19), 1.57, 1.27 (2H, s, H-21), 1.57, 1.27 (2H, s, H-22), 0.78 (3H, s, H-24), 0.81 (3H, s, H-25), 0.96 (3H, s, H-23), 0.99 (3H, s, H-27), 1.05 (3H, s, H-26), 1.70 (3H, s, H-30), 3.20 (d, H-28a), 3.65 (d, H-28b), 4.58 (dd, H-29a), 4.71 (d, H-29b). ^13^C NMR (CDCl_3_) δ: 38.73 (C-1), 27.41 (C-2), 79.01 (C-3), 38.73 (C-4), 55.32 (C-5), 18.34 (C-6), 34.30 (C-7), 40.84 (C-8), 50.45 (C-9), 37.18 (C-10), 20.95 (C-11), 25.15 (C-12), 37.18 (C-13), 42.84 (C-14), 27.41 (C-15), 29.39 (C-16), 47.99 (C-17,C-19), 48.31 (C-18), 150.94 (C-20), 29.73 (C-21), 33.30 (C-22), 28.01 (C-23), 15.40 (C-24), 16.14 (C-25), 15.99 (C-26), 14.57 (C-27), 63.67 (C-28), 109.36 (C-29), 16.00 (C-30) (Figures S7–S14), all in agreement with values in the literature [39,43]. The melting point of compound **3** was 248–250 °C.

For compound **4** (m.p.175–177 °C, optical rotation [α]D +17.2°), a detailed analysis of the NMR data led to the proposed structure, confirmed by the analysis of the spectra and comparison of the NMR resonances (Figures S15–S18) with the literature data summarized in the Table 4 [40].

#### 2.3.2. Identification of the Isolated Compounds from Fruits of *Rosa beggeriana* Schrenk

**Figure 3:**
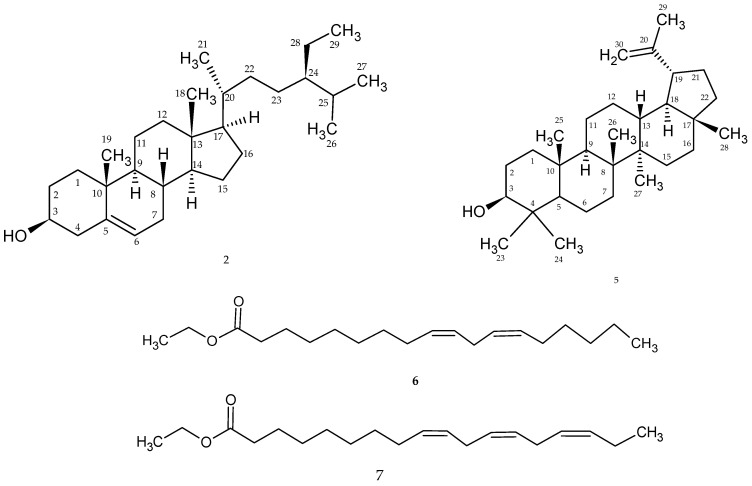
Chemical structure of β-sitosterol (**2**), lupeol (**5**), ethyl linoleate (**6**), and ethyl linolenoate (**7**).

β-sitosterol (**2**), lupeol (**5**), ethyl linoleate (**6**), and ethyl linolenoate (**7**) were isolated from the ethanol extract (35 g) of *Rosa beggeriana* Schrenk (400 g). The chemical structures are shown in Figure 3.

Compound (**2**) was identified as β-sitosterol (Figures S5 and S6) according to the literature [44,45].

Lupeol (**5**) (m.p. 215–218°C): (Figures S19–S21) ^1^H NMR (CDCl_3_) δ: 0.77 (3H, s, H-24), 0.80 (3H, s, H-28), 0.84 (3H, s, H-25), 0.96 (3H, s, H-23), 0.98 (3H, s, H-27), 1.04 (3H, s, H-26), 1.69 (3H, s, H-30), 4.58 (1H, s, H-29a), 4.70 (1H, s, H-29b). 3.19 (1H, dd, H-3); ^13^C NMR (CDCl_3_, 400 MHz): δ 150.92 (C-20), 109.37 (C-29), 78.99 (C-3), 55.32 (C-5), 50.45 (C-9), 48.31 (C-18), 47.99 (C-19), 43.01 (C-17), 42.84 (C-14), 40.84 (C-8), 40.02 (C-22), 38.87 (C-13), 38.73 (C-4), 38.06 (C-1), 37.18 (C-10), 35.60 (C-16), 34.30 (C-7), 29.86 (C-21), 28.01 (C-23), 27.46 (C-15), 27.40 (C-12), 25.15 (C-2), 20.95 (C-11), 19.33 (C-30), 18.34 (C-6), 18.03 (C-28), 16.14 (C-25), 16.00 (C-26), 15.40 (C-24), 14.57 (C-27) [39,41,46,47]. Optical rotation [α]D +27.1°.

The analysis of the NMR spectra (Figures S22–S28) and the comparison of the spectroscopic data with those described in the literature allowed the identification of the mixture of two fatty acids known as ethyl linoleate (**6**) and ethyl linolenoate (**7**), and GC-MS helped to verify the accuracy of our assumptions. ^1^H NMR (CDCl_3_) δ: 2.30 (2H, t, H-2), 1.63 (2H, t, H-3), 1.35 (14H, s, H-4, 5, 6, 7, 15, 16, 17), 2.07 (4H, m, H-8, 14), 5.41— 5.30 (4H, m, H-9, 10, 12, 13), 2.77 (2H, t, J_7.0 Hz, H-11), 5.37 (4H, m, H-9, 10, 12, 13), 4.14 (2H, m, –OCH_2_–), 0.90 (3H, t, H-18) 1.26 (3H, m, H-20). ^13^C NMR: 174.06 (C-1), 33.76 (C-2) 25.21 (C-3) 26.84(C-4) 29.32 (C-5) 29.45 (C-6) 29.45 (C-7) 27.84 (C-8) 131.36(C-9) 129.49 (C-10) 25.81 (C-11) 129.56(C-12) 129.69 (C-13), 28.81(C-14) 29.45 (C-15) 31.74 (C-16) 22.40 (C-17) 59.97 (–OCH_2_–) 13.24 (C-18) 14.35 (C-20) [48].

## 3. Discussion

### 3.1. GC-MS Data

The GC-MS analysis uncovered a heterogeneous chemical composition encompassing various classes of volatile compounds, which has been meticulously documented and organized in Table 1 and Table 2. Considering the paucity of scholarly investigations on the phytochemical composition of *R. beggeriana*, the GC-MS data were juxtaposed with data obtained from other species belonging to the *Rosa* genus, as cited in [49,50,51,52]. The comparative analysis of compositions, specifically the leaves and fruits, revealed a higher degree of complexity in the composition of the latter. It is noteworthy that this study represents the inaugural examination of the fatty acids’ profiles pertaining to this plant species.

Table 1 presents the GC-MS data in the analysis of fractions L-2-1, L-2-11, L-2-27, CH-21, CH-39, and HIJK obtained from leaves of *R. beggeriana*. The richest one was L-2-11—n-hexane/ethyl acetate fraction (6/4).

The composition of leaves was found to include various compounds such as terpenoids, specifically (-)-aristolene, as well as phytosterols like stigmastan-3,5-diene. Additionally, saturated and unsaturated fatty acids, along with their corresponding esters, were identified. The majority of the fatty acids detected exhibited unsaturation, including 9-hexadecenoic acid methyl ester (Z)-, 9,12-octadecadienoic acid (Z,Z)-methyl ester, 9,12,15-octadecatrienoic acid methyl ester (Z,Z,Z)-, methyl linoleate, methyl linolenate, methyl elaidate, 9-octadecenoic acid methyl ester (E)-, 9-octadecenoic acid (Z)- methyl ester, 9,12,15-octadecatrienoic acid (Z,Z,Z)-, and cis-13-eicosenoic acid methyl ester.

The GC-MS data obtained from the analysis of ethanol extract (B) and various fractions (B-1, B-2, B-3, B-4, B-5, B-DCM, B-M1-16, B-M2-18, 26-A, 26-S8) derived from *R. beggeriana* fruits are presented in Table 2. Among these fractions, the most abundant one was B-4, which corresponded to the chloroform/ethyl acetate fraction with a ratio of 1:1.

The fruits of the plant exhibited a comprehensive array of both saturated and unsaturated fatty acids, along with their respective esters. Moreover, several additional fatty acids were identified, including myristic acid, palmitic acid, palmitoleic acid, ethyl linoleate, (e)-9-octadecenoic acid ethyl ester, ethyl oleate, alpha-linolenic acid, 11-octadecenoic acid (z)-, 11-octadecenoic acid (e)-, oleic acid (z)-, stearic acid, 9,12-octadecadienoic acid (z,z)-, and 2-hydroxy-1-(hydroxymethyl)ethyl ester. The abundance of unsaturated fatty acids in the composition of the fruits holds significant potential for application in the food industry.

In addition, the fruits were found to encompass phytosterols such as beta-sitosterol, gamma-sitosterol, stigmastan-3,5-diene, gamma-tocopherol, and lupeol.

Upon comprehensive analysis of the collective findings presented in Table 2 and Table 3, it becomes evident that both the leaves and fruits of *R. beggeriana* exhibited a substantial abundance of saturated and unsaturated acids, terpenoids, and various other substances. These results collectively indicate a highly diverse and rich composition within the examined plant components.

### 3.2. Isolation and Identification of Individual Compounds

Identification of the isolated compounds (1–5) was based on spectroscopic analyses (^1^H, DEPTQ, DEPT135, ^13^C NMR) compared with the data published in the literature.

In this study, the presence of 3β,23-dihydroxyurs-12-ene (compound **1**—white and crystalline) in the leaves of *R. beggeriana* was established and confirmed using NMR data analysis, coupled with relevant literature sources [35,36,37]. A structurally similar compound, 3β,24-dihydroxyurs-12-ene, had been previously isolated from *Protium heptaphyllum*. By comparing the ^1^H- and ^13^C-NMR spectra of the two compounds, it was observed that all peaks were identical, except for those corresponding to C-23 and C-24. For 3β,23-dihydroxyurs-12-ene, the chemical shifts for C-23 and C-24 were 63.0 and 15.6 ppm, respectively, whereas for 3β,24-dihydroxyurs-12-ene, these values were reversed [35]. The presence of an oxygen moiety at C-23 in 3β,23-dihydroxyurs-12-ene was deduced based on the chemical shift at 63.0 ppm. The ^1^H-NMR spectrum displayed signals corresponding to seven methyl groups: 1.25 (H-24), 0.98 (H-25), 1.02 (H-26), 1.08 (H-27), 0.81 (H-28), 0.80 (H-29), and 0.81 (H-30). The ^13^C-NMR spectrum exhibited two peaks at 124.4 (C-12) and 139.5 (C-13), indicating the presence of a double bond in the ring. Overall, the number of observed peaks suggested the presence of 30 carbon atoms in compound 1. Importantly, this study represents the first isolation of 3β,23-dihydroxyurs-12-ene from the *Rosa* genus. Compound **1** was further compared to 3β,28-dihydroxyurs-12-ene, which contained a -CH_2_OH group at the 28th carbon atom, resulting in a chemical shift of 69.20 ppm. Consistent correlations were observed in the ^1^H-NMR spectra, supporting the structural analysis. High-resolution mass spectrometry (HR MS), as depicted in Figure S4, furnishes valuable insights pertaining to the molecular attributes of the compound under investigation. Specifically, it elucidated a molecular weight of 442 *m*/*z*, thereby affording a comprehensive breakdown of constituent particles within this compound. For instance, it is reasonable to deduce the presence of two highly mobile hydrogen atoms (*m*/*z* 440). Furthermore, the observation of protonation events at 424 [M + H_2_O]^+^ and 406 [M + H_2_O]^+^ suggests the existence of two hydroxyl (-OH) groups.

In addition, the HR MS data imitate the potential stability of a fragment with an *m*/*z* value of 273, indicative of a cleavage point that partitions the molecule into two relatively stable subunits. Moreover, the ensuing particles predominantly originated from the cleavage of the molecule at the central region of its third ring, yielding fragments with *m*/*z* values of 133, 189, 203, and 234. These findings contribute to a more intricate understanding of the compound’s structural composition and fragmentation pattern. Notably, structurally related compounds (although not identical) [37,43,53,54] have been identified within the *Rosa* family, specifically in the fruits of *R. multiflora* and the roots of *R. taiwanensis*, and have been associated with anti-inflammatory activity. Based on the available data, it is plausible to postulate that 3β,23-dihydroxyurs-12-ene may possess similar activities to those of the structurally related compounds mentioned earlier. However, further investigation and experimental studies are necessary to validate and ascertain its potential biological activities. This compound have potential for use in medicine because there many studies of almost structurally identical compounds that have different types of activities [37,55,56].

Betulin (**3**) and (+)-catechin (**4**) have been identified in various *Rosa* species, present not only in leaves, but also in the roots, stems, with (+)-catechin present in the fruits and flowers [43,57,58,59]. The identification of compound **3** (betulin—solid and white crystalline) was accomplished through the analysis of its characteristic ^1^H NMR (400 MHz; CDCl_3_) and ^13^C NMR (CDCl_3_) spectra. The carbon peaks were meticulously examined, revealing the presence of 30 carbon atoms in this compound. Utilizing the DEPT135 method, it was determined that betulin comprises 12 -CH_2_ groups, 5 unhydrolyzed carbons (because we can see them in ^13^C NMR spectra but not there), 13 -CH_3_ and/or -CH groups, two oxygenated carbons (63.06 and 79.01), and one double bond (109.35). Proton magnetic resonance spectra further confirmed these structural features. Specifically, the ^1^H NMR spectra exhibited signals corresponding to six methyl groups: 0.78 (3H, s, H-24), 0.81 (3H, s, H-25), 0.96 (3H, s, H-23), 0.99 (3H, s, H-27), 1.05 (3H, s, H-26), and 1.70 (3H, s, H-30). Additionally, signals of methylene groups were observed at 1.57, 1.27 (2H, s, H-1), 1.70 (2H, s, H-2), 3.21 (2H, dd, H-2), 1.38 (2H, s, H-6), 1.57, 1.38 (2H, s, H-7), 1.38 (2H, s, H-11), 1.38 (2H, s, H-12), 1.57, 1.27 (2H, s, H-15), 1.57, 1.27 (2H, s, H-16), 1.57, 1.27 (2H, s, H-21), 1.57, and 1.27 (2H, s, H-22). Based on the data presented in Figures S10–S14, encompassing 2D nuclear magnetic resonance (NMR) and gas chromatography-mass spectrometry (GC-MS) spectra, it is discernible that compound **3** corresponds to betulin. The GC-MS spectra provides essential information such as the retention time and molecular weight of this compound. Additionally, compound (+)-catechin (**4**) has been identified through a combination of NMR analysis, which was previously elucidated, and mass spectrometry. The mass spectra of compound **4** exhibited a discernible molecular weight of approximately 290 units. This determination is in accordance with our NMR-based assumption of it being catechin. Notably, the mass spectra also revealed distinct ions with molecular weights of 110, 138, and 55 units. These observations are congruent with the structural features of catechin, particularly with regard to the presence of vulnerable chemical bonds in its structure. Betulin was isolated from the roots of *Rosa taiwanensis* [43], and catechin was reported in the roots of *R. taiwanensis* [43], rosehips of *Rosa canina* [57,58]. Notably, betulin was reported to have anti-inflammatory and anticancer properties [60]. The structural identification of (+)-catechin (4) was established based on the analyses of the ^1^H NMR, DEPT135, and ^13^C NMR signals.

The NMR data of β-sitosterol (**2**) were subjected to a comprehensive comparison with relevant literature data [44,45,61].

Lupeol (**5**) is a triterpenoid that was found in the fruits of *R. beggeriana* and identified through comparison with NMR data from the literature data [39,46,47]. *R. rugosa* was also reported to have lupeol [62]. Using carbon nuclear magnetic resonance, it was found that the compound had 30 carbon atoms, 10 of which were methylene groups. The presence of a hydroxyl group and a double bond due to the presence of a shift was also established. Also, thanks to proton magnetic resonance, it was possible to establish the structure by calculating the number and area of peaks.

Ethyl linoleate (**6**) and ethyl linolenoate (**7**) were isolated together, and structure was elucidated by GC-MS data (Figures S21 and S22) and NMR, which were compared to literature [48].

Regardless of the precise study of several species, like *R. canina*, *R. rugosa*, and others, there are many species in the *Rosa* genus with minimal data. Many species of the *Rosa* family grow in diverse conditions, hence they can have different chemical composition [1,4,5].

## 4. Materials and Methods

### 4.1. Plant Material

The plant was harvested at a 23–25 °C temperature near Ili River and in the Almaty oblast N44°79.3959, E76°29.8245 in September 2021, by a biologist employee of the Botanical Garden in Almaty, Madina Ramazanova. Then, the plant was dried in the drying cabinet at 35 °C for 6 h and 48 h at room temperature, And it was deposited at the herbarium collection at the Institute of Botany and Phytointroduction of the Ministry of Higher Education and Science, Almaty, Kazakhstan (0002540).

### 4.2. General Experimental Procedures

Solvents used in this work, n-hexane, chloroform, dichloromethane (DCM), ethyl acetate (EtOAc), methanol (MeOH), and ethanol (EtOH), were purchased from Fisher Scientific, Waltham, MA USA. Deuterated solvents (Sigma-Aldrich, Darmstadt, Germany), including methanol (MeOD) and chloroform (CDCl_3_), were used for nuclear magnetic resonance (NMR) spectroscopic analyses. Column chromatography (CC) was performed using silica gel 60 (0.063–0.200 mm; Merck, Darmstadt, Germany) or Sephadex LH-20 (0.25–0.1 mm, GE Healthcare, Cytiva, Sweden). Vacuum liquid chromatography column (VLC) (diameter 15 cm × length 30 cm, 300 g) at room temperature was used to isolate substances from five main fractions. Thin-layer chromatography (TLC) analyses were carried out using pre-coated silica G plates w/UV254 (20 cm × 20 cm, 200 µm in thickness; Sorbent Technologies, Norcross, GA, USA). An ultraviolet lamp (UVP, LLC, Spectroline, Westbury, NY, USA) was used for the visualization of spots on thin-layer chromatograms at 254 and/or 365 nm. Spots were visualized by spraying with 2% vanillin–sulfuric acid in ethanol followed by heating at 110 °C on a hot plate. Moreover, ^1^H, DEPT135, DEPTQ and ^13^C NMR spectra were recorded on a Bruker Avance 400 MHz instrument (Bruker, MA, USA). An LTQ Orbitrap XL mass spectrometer (Agilent Technologies, Santa Clara, CA, USA) was used for high-resolution-electrospray ionization-mass spectrum (HR-ESI-MS). The GC-MS analysis was performed with a Agilent 7890A gas chromatograph (Agilent Technologies, Santa Clara, CA, USA) coupled with an Agilent 5975C single quadrupole mass spectrometer (Agilent Technologies, Santa Clara, CA, USA).

### 4.3. Extraction and Isolation

#### 4.3.1. Extraction and Isolation of Leaves

The air-dried leaves (553.0 g) were macerated with ethanol 95% (1.5 L × 3 times) at room temperature. The ethanol extracts were combined, and the solvent was distilled under reduced pressure at low temperature to afford a 10.0 g yield.

The ethanol extract was processed using vacuum liquid chromatographic techniques with silica gel in a column (600 g, 0.063–0.200 mm; Merck, Darmstadt, Germany). The extract was eluted using a gradient system with n-hexane, DCM, ethyl acetate, and ethanol, with growing polarity as a mobile phase starting with 100% n-hexane and ending with 100% methanol, and 5 fractions were obtained. Fractions were grouped depending on their chemical similarity and monitored using thin-layer chromatography and concentrated using a rotary evaporator. The obtained fractions were L-F1 (1.35 g), L-F2 (0.56 g), L-F3 (0.74 g), L-F4 (1.85 g), and L-F5 (2.37 g).

L-F1, L-F2, and L-F3 were concentrated together to fraction L-F1 according to similar spots on TLC. L-F4 and L-F5 was collected to fraction L-F2. Then, each fraction was separated using chromatographic fractionation in a glass column with silica gel 60 (200 g, 0.063–0.200 mm; Merck, Darmstadt, Germany) and Sephadex LH-20 (Lipophilic, 25–100 µm, Sigma). The mobile phase was n-hexane and ethyl acetate, and 12 fractions were obtained.

Some fractions that were isolated from fractions L-F1 and L-F2 were studied by GC-MS. To analyze fatty acids by GC-MS, it was prepared by refluxing 20 mg of the isolated fractions with 20 mL CH_3_OH and 2 mL H_2_SO_4_ for 4 h.

#### 4.3.2. Extraction and Isolation of Fruits

The air-dried fruits (400.0 g) were macerated with ethanol 95% (1.5 L ×3 times) at room temperature. The ethanol extracts were combined, and the solvent was distilled under reduced pressure at low temperature to afford a 15.0 g yield.

The ethanol extract was processed using vacuum liquid chromatographic techniques with silica gel premium-grade C18 (40–63 µm; 60Å; Sorbtech, Norcross GA, USA) in a column. The extract was eluted using a gradient system with DCM, methanol, and water, with decreasing polarity as a mobile phase starting with 100% water and ending with 100% DCM, and 6 fractions were obtained.

Fractions were grouped depending on their chemical similarity and monitored using thin-layer chromatography and concentrated using a rotary evaporator. The obtained fractions were B-F1 (2.32 g), B-F2 (2.56 g), B-F3 (2.15 g), B-F4 (1.85 g), and B-F5 (1.37 g).

Subsequently, the fractions were separated on Sephadex LH-20 (0.25–0.1 mm, GE Healthcare, Sweden) using methanol as the eluent. As a result, compounds 2, 5, 6, and 7 were obtained.

Some fractions that were isolated from fractions B-F1, B-F2, B-F3, B-F4, and B-F5 were studied by GC-MS. To analyze fatty acids by GC-MS, it was prepared by refluxing 20 mg of the isolated fractions with 20 mL CH_3_OH and 2 mL H_2_SO_4_ for 4 h.

## 5. Conclusions

Previously, this type of rosehip (*Rosa beggeriana* Schrenk) had not been studied in terms of chemical composition, hence there are no articles on isolated compounds from this plant. However, there are many research articles about species from the genus *Rosa*, which are very well known in traditional medicine. The fractionation of *Rosa beggeriana* Schrenk leaves and fruits resulted in the isolation and structural elucidation of seven compounds, including phytosterol, triterpenoids, polyphenol, and mixture of fatty acids. β-sitosterol (**2**), betulin (**3**), (+)-catechin (**4**), lupeol (**5**), ethyl linoleate (**6**) have already been isolated from the genus *Rosa* but not from *Rosa beggeriana* Schrenk. And compounds like 3β,23-dihydroxyurs-12-ene (**1**) and ethyl linolenoate (**7**) were isolated for the first time for both *Rosa* and *Rosa beggeriana* Schrenk.

## Figures and Tables

**Table 1:** GC-MS data for the leaves of *Rosa beggeriana* Schrenk.

RT	Compound Name	Match Factor	Area, %
L-2-1—Hexane fraction			
40.944	Octadeca-9,12,15-trienoic acid	95.8	5.38
L-2-11—n-hexane/ethyl acetate fraction (6/4)			
28.259	Methyl dodecanoate	98.0	0.95
32.897	Methyl tetradecanoate	97.5	2.61
37.087	Methyl hexadecanoate	98.6	14.45
39.021	Methyl heptadecanoate	95.0	0.47
40.244	Methyl octadeca-9,12-dienoate	99.2	13.16
40.335	Methyl octadeca-9,12,15-trienoate	99.3	32.94
40,387	Methyl trans-9-octadecenoate	91.9	5.86
40.483	Methyl (9Z)-9-octadecenoate	96.5	3.06
40.878	Methyl octadecanoate	98.6	6.37
44.340	Methyl icosanoate	96.2	1.92
47.540	Methyl docosanoate	95.1	2.16
51.111	Methyl tetracosanoate	95.3	1.45
55.902	Methyl Hexacosanoate	90.3	1.67
CH-21—dichloromethane/ethyl acetate fraction (7/3)			
37.078	Methyl hexadecanoate	90.4	5.04
CH-39—dichloromethane/ethyl acetate fraction (6/4)			
14.411	2-Ethylhexan-1-ol	96.4	1.85
32.897	Methyl tetradecanoate	96.3	4.83
37.078	Methyl hexadecanoate	93.1	2.00
40.225	Methyl octadeca-9,12-dienoate	96.3	3.76
40.297	Methyl octadeca-9,12,15-trienoate	97.6	9.75
28.259	Methyl dodecanoate	96.6	0.58
35.521	6,10,14-Trimethylpentadecan-2-one	96.3	0.58
35.649	7,11,15-Trimethyl-3-methylidenehexadec-1-ene	92.9	0.33
36.125	(2E,7R,11R)-3,7,11,15-Tetramethylhexadec-2-en-1-yl acetate	92.3	0.53
36.502	3,7,11,15-Tetramethyl-2-hexadecen-1-ol	93.2	2.10
37.078	Methyl hexadecanoate	98.4	12.43
40.302	Methyl octadeca-9,12,15-trienoate	99.2	4.85
40.874	Methyl octadecanoate	96.8	13.58
41.440	Ethyl (9Z,12Z)-octadeca-9,12-dienoate	94.6	14.49
44.340	Methyl icosanoate	90.1	10.66
L-2-27—ethyl acetate/methanol fraction (8/2)			
37.697	Hexadecanoic acid	94.1	6.28
40.926	Octadeca-9,12,15-trienoic acid	93.1	7.28

**Table 2:** GC-MS data for the fruits of *Rosa beggeriana Schrenk*.

RT	Compound Name	Match Factor	Area, %
B—Ethanol extract			
26.198	Trimethyl 2-hydroxybutane-1,2,3-tricarboxylate	91.7	13.47
39.397	Ethyl hexadecanoate	92.4	5.25
42.430	Ethyl (9E,12E)-octadeca-9,12-dienoate	92.5	6.71
42.506	Ethyl (9E,12E,15E)-octadeca-9,12,15-trienoate	90.0	6.55
39.397	Ethyl hexadecanoate	92.7	4.97
42.430	Ethyl (9E,12E)-octadeca-9,12-dienoate	92.6	6.47
42.506	Ethyl (9E,12E,15E)-octadeca-9,12,15-trienoate	91.6	6.29
42.430	Ethyl (9Z,12Z,15Z)-octadeca-9,12,15-trienoate	91.2	3.55
38.068	Methyl hexadecanoate	96.6	4.32
38.959	Ethyl 9-hexadecenoate	92.1	0.83
41.216	Methyl octadeca-9,12-dienoate	99.0	11.16
41.292	Methyl octadeca-9,12,15-trienoate	98.0	8.22
41.364	Methyl (9Z)-9-octadecenoate	93.5	4.57
41.864	Methyl octadecanoate	90.8	1.53
42.435	Ethyl (9Z,12Z,15Z)-octadeca-9,12,15-trienoate	97.9	15.56
42.516	Ethyl (9E,12E,15E)-octadeca-9,12,15-trienoate	98.3	11.37
42.573	Ethyl (E)-octadec-9-enoate	93.2	6.22
43.059	Ethyl octadecanoate	93.8	1.50
42.425	Ethyl (9Z,12Z)-octadeca-9,12-dienoate	90.6	4.82
42.430	Ethyl (9Z,12Z,15Z)-octadeca-9,12,15-trienoate	92.8	3.09
48.811	14-(5-Ethyl-6-methylheptan-2-yl)-2,15-dimethyltetracyclo [8.7.0.0^{2,7}.0^{11,15}]heptadec-7-en-5-ol	93.9	30.29
56.445	Nonacosane	92.1	13.69
B-M1-16—ethyl acetate/methanol fraction (1/1)			
37.621	Methyl (Z)-pentadec-8-enoate	96.1	0.85
38.073	Methyl hexadecanoate	98.5	4.23
38.968	Ethyl 9-hexadecenoate	97.9	1.81
39.402	Ethyl hexadecanoate	98.7	7.97
41.221	Methyl octadeca-9,12-dienoate	99.2	5.82
41.297	Methyl octadeca-9,12,15-trienoate	98.8	7.00
41.373	Methyl (9Z)-9-octadecenoate	93.0	2.84
41.873	Methyl octadecanoate	92.2	0.53
42.449	Ethyl (9Z,12Z,15Z)-octadeca-9,12,15-trienoate	98.4	27.71
42.530	Ethyl (9E,12E,15E)-octadeca-9,12,15-trienoate	98.8	28.61
43.068	Ethyl octadecanoate	93.2	0.80
60.378	(2R)-2,7,8-Trimethyl-2-[(4R,8R)-4,8,12-trimethyltridecyl]-3,4-dihydro-2H-1-benzopyran-6-ol	91.4	1.66
B-M2-18—ethyl acetate/methanol fraction (3/7)			
32.478	Methyl dodecanoate	96.9	0.61
36.706	Tetradecanoic acid	99.6	3.22
38.321	(9Z)-Hexadec-9-enoic acid	97.9	3.81
38.854	Hexadecanoic acid	98.1	29.50
42.002	(9Z,12Z)-Octadeca-9,12-dienoic acid	97.7	27.43
42.135	(E)-Octadec-9-enoic acid	97.4	23.95
42.492	(E)-Octadec-2-enoic acid	96.6	3.19
26-A—ethyl acetate/methanol fraction (2/8)			
40.549	Hexadecanoic acid	95.8	8.32
40.549	Hexadecanoic acid	98.6	29.85
43.535	(9Z,12Z,15Z)-Octadeca-9,12,15-trienoic acid	91.1	3.47
62.859	(8S,9S,10R,13R,14S,17R)-17-[(2R,5R)-5-Ethyl-6-methylheptan-2-yl]-10,13-dimethyl-2,7,8,9,11,12,14,15,16,17-decahydro-1H-cyclopenta[a]phenanthrene	90.0	6.04

**Table 3:** Spectral data of ^1^H NMR and ^13^C NMR of 3β,23-dihydroxyurs-12-ene (**1**) in CDCl_3_ and the structures of similar molecules.

No.	^13^C NMR Compound 1	Ref. ^13^C NMR (3β,24-Dihydroxyurs-12-ene) [35]	Ref. ^13^C NMR (3β,28-Dihydroxyurs-12-ene) [36]	^1^H NMR Compound 1	Ref. ^1^H NMR (3β,24-Dihydroxyurs-12-ene) [35]	Ref. ^1^H NMR (3β,28-Dihydroxyurs-12-ene) [36]
1	38.3	38.5	38.78		-	
2	27.3	27.2	26.63		-	
3	79.0	80.9	78.44	3.32, m	3.45 dd, 11.5/4.4 Hz	3.17
4	42.0	42.0	38.60	-	-	-
5	55.2	55.8	55.15		-	
6	18.4	18.6	18.20		-	
7	32.9	33.1	32.72		-	
8	40.0	40.0	39.86	-	-	-
9	47.7	47.7	47.56		-	
10	36.9	36.6	36.75	-	-	-
11	23.4	23.6	23.22		-	
12	124.4	124.2	124.96	5.26, m	5.12 br t, 3.6 Hz	5.1
13	139.5	139.6	138.67	-	-	-
14	42.7	42.7	42.32	-	-	-
15	28.1	28.0	26.24		-	
16	26.6	26.6	23.70		-	
17	33.7	33.7	37.79	-	-	-
18	59.1	59.0	54.07	-	-	-
19	39.6	39.5	39.62		-	
20	39.7	39.6	39.33		-	
21	31.2	31.2	30.80		-	
22	41.5	41.5	35.15		-	
23	63.0	22.4	27.89	3.72, m	1.25	0.80
24	15.6	64.5	16.44	1.33, s	4.23/3.34 d,11.0 Hz	0.93
25	15.7	16.2	15.51	1.06, s	0.90	0.92
26	16.9	16.7	16.44	1.10, s	0.97	0.92
27	23.3	23.3	23.70	1.16, s	1.06	1.09
28	28.8	28.7	69.20	0.89, s	0.79	3.52
29	17.5	17.5	17.23	0.88, s	0.78	0.81
30	21.4	21.4	21.20	0.89, s	0.91	1.00

**Table 4:** Spectral data of ^1^H NMR and ^13^C NMR of (+)-catechin (**4**) in MeOD and chemical structures of the molecules.

No.	^13^C NMR	Ref. ^13^C NMR	^1^H NMR
2	82.9	83.0	4.59 (d, J = 7.44 Hz)
3	68.9	68.9	4.00 (q, J = 8.2 Hz)
4	28.6	28.6	2.88 (dd, J = 16.12, 5.36 Hz) 2.52 (dd, J = 16.08, 8.08 Hz)
5	157.6	157.7	
6	96.5	96.4	5.89 (d, J = 2.3 Hz)
7	157.9	157.9	
8	95.7	95.6	5.96 (d, J = 2.3 Hz)
9	157.0	157.0	
10	101.0	100.9	
1′	132.3	132.3	
2′	115.4	115.4	6.86 (d, J = 1.96 Hz)
3′	146.3	146.4	
4′	146.3	146.3	
5′	116.3	116.2	6.78 (d, J = 8.16 Hz)
6′	120.2	120.2	6.74 (dd, J = 8.16, 2.0 Hz)

## Data Availability

Not applicable.

## Supplementary Materials

The following supporting information can be downloaded at: https://www.mdpi.com/article/10.3390/plants12183297/s1, Figure S1: ^1^H NMR spectrum of 3β,23-Dihydroxyurs-12-ene (**1**); Figure S2: DEPT135 NMR spectrum of 3β,23-Dihydroxyurs-12-ene (**1**); Figure S3: ^13^C NMR spectrum of 3β,23-Dihydroxyurs-12-ene (**1**); Figure S4: Mass spectrum of 3β,23-Dihydroxyurs-12-ene (**1**); Figure S5: ^1^H NMR spectrum of β-sitosterol (**2**); Figure S6: DEPTQ NMR spectrum of β-sitosterol (**2**); Figure S7: ^1^H NMR spectrum of Betulin (**2**); Figure S8: DEPT135 NMR spectrum of Betulin (**2**); Figure S9: ^13^C NMR spectrum of Betulin (**2**); Figure S10: HSQC NMR spectrum of Betulin (**2**); Figure S11: HSQC NMR spectrum of Betulin (**2**); Figure S12: HSQC NMR spectrum of Betulin (**2**); Figure S13: HSQC NMR spectrum of Betulin (**2**); Figure S14: GC-MS spectrum of Betulin (**2**); Figure S15: ^1^H NMR spectrum of (+)-Catechin (**3**); Figure S16: DEPT NMR spectrum (+)-Catechin (**3**); Figure S17: ^13^C NMR spectrum of (+)-Catechin (**3**); Figure S18: Mass spectrum of (+)-Catechin (**3**); Figure S19: ^1^H NMR spectrum of Lupeol (**4**); Figure S20: DEPT135 NMR spectrum of Lupeol (**4**); Figure S21: GC-MS Data of Lupeol (**4**); Figure S22: ^1^H NMR spectrum of Ehyl linoleate (**5**) and Ethyl linolenoate (**6**); Figure S23: ^1^H NMR spectrum of Ehyl linoleate (**5**) and Ethyl linolenoate (**6**); Figure S24: DEPT135 NMR spectrum of Ehyl linoleate (**5**) and Ethyl linolenoate (**6**); Figure S25: ^13^C NMR spectrum of Ehyl linoleate (**5**) and Ethyl linolenoate (**6**); Figure S26: GC-MS Data of Ethyl linoleate (**5**) and Ethyl linolenoate (**6**); Figure S27: GC-MS Data of Ethyl linolenoate(**6**); Figure S28: GC-MS Data of Ethyl linoleate(**5**).

## References

[1] Leus L., Van Laere K., De Riek J., Van Huylenbroeck J., Van Huylenbroeck J. (2018). Chapter 27. Rose. Ornamental Crops. Handbook of Plant Breeding.

[2] Fougère-Danezan M., Joly S., Bruneau A., Gao X.F., Zhang L.B. (2015). Phylogeny and Biogeography of Wild Roses with Specific Attention to Polyploids. Ann. Bot..

[3] Ibrahimov A.M., Talibov T.H., Matsyura A.V. (2018). The *Genus Rosa* L. (Rosaceae) in the Flora of Nakhchivan Autonomous Republic (Azerbaijan). Acta Biol. Sib..

[4] Handa S.S., Rakesh D.D., Vasisht K.J. (2006). Compendium of Medicinal and Aromatic Plants ASIA.

[5] Ayati Z., Amiri M.S., Ramezani M., Delshad E., Sahebkar A., Emami S.A. (2018). Phytochemistry, Traditional Uses and Pharmacological Profile of Rose Hip: A Review. Curr. Pharm. Des..

[6] Ginko E., Alajmovic Demirović E., Šarić-Kundalić B. (2023). Ethnobotanical Study of Traditionally Used Plants in the Municipality of Zavidovići, BiH. J. Ethnopharmacol..

[7] Liu T., Lu Y., Tonissen K., Di Trapani G., Tang W., Feng Y. (2022). Application of Traditional Chinese Medicine as Skin Depigmentation Agents. Heliyon.

[8] Kreidel M.K., Jhaveri M. (2021). Introduction to Essential Oils and Essential Oil Processing. Integrative Dermatology: Practical Applications in Acne and Rosacea.

[9] Jasim Z.M., Jasim G.A., Abbas I.S. (2022). Anti-Angiogenic Activity of Rosa Canina Extracts, an Ex-Vivo and In-Vivo Study. Int. J. Drug Deliv. Technol..

[10] Ko C.Y., Chao J., Chen P.Y., Su S.Y., Maeda T., Lin C.Y., Chiang H.C., Huang S.S. (2021). Ethnobotanical Survey on Skin Whitening Prescriptions of Traditional Chinese Medicine in Taiwan. Front. Pharmacol..

[11] Sargin S.A. (2021). Potential Anti-Influenza Effective Plants Used in Turkish Folk Medicine: A Review. J. Ethnopharmacol..

[12] Sallustio V., Chiocchio I., Mandrone M., Cirrincione M., Protti M., Farruggia G., Abruzzo A., Luppi B., Bigucci F., Mercolini L. (2022). Extraction, Encapsulation into Lipid Vesicular Systems, and Biological Activity of *Rosa canina* L. Bioactive Compounds for Dermocosmetic Use. Molecules.

[13] Upman K., Sharma A. (2023). Ethnobotany, Phytochemistry, Pharmacology, and Toxicology of Rosa Sericea a Medicinal Plant. IOP Conf. Ser. Earth Environ. Sci..

[14] Mileva M., Ilieva Y., Jovtchev G., Gateva S., Zaharieva M.M., Georgieva A., Dimitrova L., Dobreva A., Angelova T., Vilhelmova-Ilieva N. (2021). Rose Flowers—A Delicate Perfume or a Natural Healer?. Biomolecules.

[15] Wang Y., Zhao Y., Liu X., Li J., Zhang J., Liu D. (2022). Chemical Constituents and Pharmacological Activities of Medicinal Plants from *Rosa* Genus. Chin. Herb. Med..

[16] Dini S., Chen Q., Fatemi F., Asri Y. (2022). Phytochemical and Biological Activities of Some Iranian Medicinal Plants. Pharm. Biol..

[17] Li B.-L., Yuan J., Wu J.-W. (2021). A Review on the Phytochemical and Pharmacological Properties of *Rosa laevigata*: A Medicinal and Edible Plant. Chem. Pharm. Bull..

[18] Patel S. (2017). Rose Hip as an Underutilized Functional Food: Evidence-Based Review. Trends Food Sci. Technol..

[19] Jiménez S., Jiménez-Moreno N., Luquin A., Laguna M., Rodríguez-Yoldi M.J., Ancín-Azpilicueta C. (2017). Chemical Composition of Rosehips from Different *Rosa* Species: An Alternative Source of Antioxidants for the Food Industry. Food Addit. Contam. Part A Chem. Anal. Control Expo. Risk Assess..

[20] Tolekova S., Sharmanov T., Sinyavskiy Y., Berzhanova R., Mammadov R., Aksoy O.K., Yusifli R. (2020). Antioxidant, Pharmacological, Medical Properties and Chemical Content of *Rosa* L. Extracts. Int. J. Second. Metab..

[21] Quan X.X., Huang Y.Y., Chen L., Yuan J.Q. (2022). Traditional Uses, Phytochemical, Pharmacology, Quality Control and Modern Applications of Two Important Chinese Medicines from Rosa Laevigata Michx.: A Review. Front. Pharmacol..

[22] Saad B., Azaizeh H., Abu-Hijleh G., Said O. (2006). Safety of Traditional Arab Herbal Medicine. Evid.-Based Complement. Altern. Med..

[23] Ahmad N., Anwar F., Gilani A.-u.-H. (2015). Rose Hip (Rosa Canina L.) Oils.

[24] Demir F., Özcan M. (2001). Chemical and Technological Properties of Rose (*Rosa Canina* L.) Fruits Grown Wild in Turkey. J. Food Eng..

[25] Liaudanskas M., Noreikienė I., Zymonė K., Juodytė R., Žvikas V., Janulis V. (2021). Composition and Antioxidant Activity of Phenolic Compounds in Fruit of the Genus *Rosa* L.. Antioxidants.

[26] Kubczak M., Khassenova A.B., Skalski B., Michlewska S., Wielanek M., Aralbayeva A.N., Murzakhmetova M.K., Zamaraeva M., Skłodowska M., Bryszewska M. (2020). Bioactive Compounds and Antiradical Activity of the *Rosa canina* L. Leaf and Twig Extracts. Agronomy.

[27] Fetni S., Bertella N., Ouahab A., Martinez Zapater J.M., De Pascual-Teresa Fernandez S. (2020). Composition and Biological Activity of the Algerian Plant *Rosa canina* L. by HPLC-UV-MS. Arab. J. Chem..

[28] Sabitov A., Gaweł-Bęben K., Sakipova Z., Strzępek-Gomółka M., Hoian U., Satbayeva E., Głowniak K., Ludwiczuk A. (2021). *Rosa platyacantha* Schrenk from Kazakhstan—Natural Source of Bioactive Compounds with Cosmetic Significance. Molecules.

[29] Yang S.H., Wei J.J., Yan F., Jia R.D., Zhao X., Gan Y., Ge H. (2020). Differences in Leaf Anatomy, Photosynthesis, and Photoprotective Strategies in the Yellow-Green Leaf Mutant and Wild Type of *Rosa beggeriana* Schrenk. Photosynthetica.

[30] Sun Y., Zhou M., Luo L., Pan H., Zhang Q., Yu C. (2023). Metabolic Profiles, Bioactive Compounds and Antioxidant Activity of Rosehips from Xinjiang, China. LWT.

[31] Javanmard M., Asadi-Gharneh H.A., Nikneshan P. (2018). Fruit Characteristics of Wild Rose Hip (*Rosa* spp.) Genotypes from Isfahan Region of Iran. Acta Hortic..

[32] Vaezi J., Arjmandi A.A., Sharghi H.R. (2019). Origin of *Rosa* x *binaloudensis* (Rosaceae), a New Natural Hybrid Species from Iran. Phytotaxa.

[33] Sitpayeva G.T., Kudabayeva G.M., Dimeyeva L.A., Gemejiyeva N.G., Vesselova P.V. (2020). Crop Wild Relatives of Kazakhstani Tien Shan: Flora, Vegetation, Resources. Plant Divers..

[34] Zarei O., Yaghoobi M.M. (2019). Cytotoxic and Anti-Proliferative Effects of *Rosa beggeriana* Schrenk Extracts on Human Liver and Breast Cancer Cells. Avicenna J. Phytomed..

[35] Susunaga G.S., Siani A.C., Pizzolatti M.G., Yunes R.A., Delle Monache F. (2001). Triterpenes from the Resin of Protium Heptaphyllum. Fitoterapia.

[36] El-Seed H.R. (2005). Antimicrobial Triterpenes from *Poulsenia Armata* Miq. Standl. Nat. Prod. Res..

[37] Jiang H., Han H., Man W.J., Hou A.J., Guo X.Y., Xing X.D., Yan M.L., Yang L., Yang L. (2020). Ursane-Type Triterpenoids from the Roots of *Rosa multiflora* with Their Anti-Inflammatory Activity. J. Asian Nat. Prod. Res..

[38] Quradha M.M., Khan R., Adhikari A., Rauf A., Rashid U., Bawazeer S., Al-Awthan Y.S., Bahattab O., Mubarak M.S. (2021). Isolation, Biological Evaluation, and Molecular Docking Studies of Compounds from *Sophora mollis* (Royle) Graham Ex Baker. ACS Omega.

[39] Yamashita H., Matsuzaki M., Kurokawa Y., Nakane T., Lee K.H., Shibata T., Bando H., Wada K., Gakuen H.T., Carolina N. (2019). Four New Triterpenoids from the Bark of *Euonymus alatus* Forma *Ciliato-Dentatus*. Phytochem. Lett..

[40] Cren-Olivé C., Wieruszeski J.M., Maes E., Rolando C. (2002). Catechin and Epicatechin Deprotonation Followed by 13C NMR. Tetrahedron Lett..

[41] Niaz A., Adnan A., Bashir R., Mumtaz M.W., Raza S.A., Rashid U., Tan C.P., Tan T.B. (2021). The In Vitro α-Glucosidase Inhibition Activity of Various Solvent Fractions of *Tamarix dioica* and 1H-NMR Based Metabolite Identification and Molecular Docking Analysis. Plants.

[42] Mrabti H.N., Jaradat N., Fichtali I., Ouedrhiri W., Jodeh S., Ayesh S., Cherrah Y., Faouzi M.E.A. (2018). Separation, Identification, and Antidiabetic Activity of Catechin Isolated from *Arbutus unedo* L. Plant Roots. Plants.

[43] Yang S.-C., Fang J.-M., Cheng Y.-S. (1995). Chemical Constituents from the Root and Aerial Parts of *Rosa taiwanensis*. J. Chin. Chem. Soc..

[44] Pateh U.U., Haruna A.K., Garba M., Iliya I., Sule I.M., Abubakar M.S., Ambi A.A. (2009). Isolation of Stigmasterol, β-Sitosterol and 2-Hydroxyhexadecanoic Acid Methyl Ester from the Rhizomes of *Stylochiton lancifolius*, Pyer & Kotchy (Araceae). Niger. J. Pharm. Sci..

[45] Kamboj A., Saluja A.K. (2011). Isolation of Stigmasterol and β-Sitosterol from Petroleum Ether Extract of Aerial Parts of *Ageratum Conyzoides* (Asteraceae). Int. J. Pharm. Pharm. Sci..

[46] Jamal A.K., Yaacob W.A., Din L.B. (2008). A Chemical Study on Phyllanthus Reticulatus. J. Phys. Sci..

[47] Mohamed S.M., Ross S.A., Mohamed N.M. (2022). Exploration of Components Contributing to Potent Cytotoxicity of Gardenia *Thunbergia* L. F. against Human Leukemia and Hepatoma. Bull. Pharm. Sci..

[48] Huh S., Kim Y.S., Jung E., Lim J., Jung K.S., Kim M.O., Lee J., Park D. (2010). Melanogenesis Inhibitory Effect of Fatty Acid Alkyl Esters Isolated from Oxalis Triangularis. Biol. Pharm. Bull..

[49] Li C., Luo Y., Zhang W., Cai Q., Wu X., Tan Z., Chen R., Chen Z., Wang S., Zhang L. (2021). A Comparative Study on Chemical Compositions and Biological Activities of Four Essential Oils: *Cymbopogon citratus* (DC.) Stapf, *Cinnamomum cassia* (L.) Presl, *Salvia japonica* Thunb. and *Rosa rugosa* Thunb. J. Ethnopharmacol..

[50] Dobreva A., Nedeltcheva-Antonova D., Nenov N., Getchovska K., Antonov L. (2021). Subcritical Extracts from Major Species of Oil-Bearing Roses—A Comparative Chemical Profiling. Molecules.

[51] Savych A., Basaraba R., Muzyka N., Ilashchuk P. (2021). Analysis of Fatty Acid Composition Content in the Plant Components of Antidiabetic Herbal Mixture by GC-MS. Pharmacia.

[52] Öz M., Deniz I., Okan O.T., Baltaci C., Karatas S.M. (2021). Determination of the Chemical Composition, Antioxidant and Antimicrobial Activities of Different Parts of *Rosa canina* L. and *Rosa pimpinellifolia* L. Essential Oils. J. Essent. Oil-Bear. Plants.

[53] Ji Y., Xia X., Xu X., Zhu N. (2020). Three New Triterpenoids with Their Bioactives from the Roots of *Rosa cymosa*. Nat. Prod. Res..

[54] Yeo H., Park S.Y., Kim J. (1998). A-Ring Contracted Triterpenoid from *Rosa multiflora*. Phytochemistry.

[55] Lee M.K., Kim S.H., Yang H., Lim D.Y., Ryu J.H., Lee E.S., Jew S.S., Park H.G., Sung S.H., Kim Y.C. (2009). Asiatic Acid Derivatives Protect Primary Cultures of Rat Hepatocytes against Carbon Tetrachloride-Induced Injury via the Cellular Antioxidant System. Nat. Prod. Commun..

[56] Khwaza V., Mlala S., Oyedeji O.O., Aderibigbe B.A. (2021). Pentacyclic Triterpenoids with Nitrogen-Containing Heterocyclic Moiety, Privileged Hybrids in Anticancer Drug Discovery. Molecules.

[57] Ozkan G., Capanoglu E., Esatbeyoglu T. (2022). Formulation of Functional Drink with Milk Fortification: Effects on the Bioaccessibility and Intestinal Absorption of Phenolics. Plants.

[58] Sabahi Z., Hasan S.M.F., Ayatollahi S.A., Farmani F., Afsari A., Moein M. (2022). Improvement of Phenolic Compound Extraction by Using Ion Exchange Chromatography and Evaluation of Biological Activities of Polyphenol-Enriched Fraction of Rosa Canina Fruits. Iran. J. Pharm. Res..

[59] Dashbaldan S., Pączkowski C., Szakiel A. (2020). Variations in Triterpenoid Deposition in Cuticular Waxes during Development and Maturation of Selected Fruits of Rosaceae Family. Int. J. Mol. Sci..

[60] Tuli H.S., Sak K., Gupta D.S., Kaur G., Aggarwal D., Parashar N.C., Choudhary R., Yerer M.B., Kaur J., Kumar M. (2021). Anti-Inflammatory and Anticancer Properties of Birch Bark-Derived Betulin: Recent Developments. Plants.

[61] Mihalcea L., Păcularu-Burada B., Milea Ș.-A., Aprodu I., Condurache (Lazăr) N.-N., Cucolea E.I., Dănilă G.-M., Cîrciumaru A., Nicoleta S. (2023). CO_2_ Supercritical Extraction and Microencapsulation of Oleoresins from Rosehip Fruits for Getting Powders with Multiple Applications. Curr. Res. Food Sci..

[62] Wang J., Wang P., Xu M., Chen Y., Feng L. (2022). Systematic Identification and Analysis of OSC Gene Family of *Rosa rugosa* Thunb. Int. J. Mol. Sci..

