# Supplementary material for: Study of the Chemical Composition of Rosa beggeriana Schrenk’s Fruits and Leaves
Source: Plants (Basel). 2023 Sep 18;12(18):3297. doi: 10.3390/plants12183297 (PMC10536339; doi:10.3390/plants12183297)
Supplement: Supplementary file 1 [file plants-12-03297-s001.zip › plants-2517924-supplementary.pdf]

Article

Study of chemical composition of *Rosa beggeriana* Schrenk's fruits and leaves

Aigerim Aituarova, Galiya E. Zhusupova, Aizhan Zhussupova and Samir A. Ross

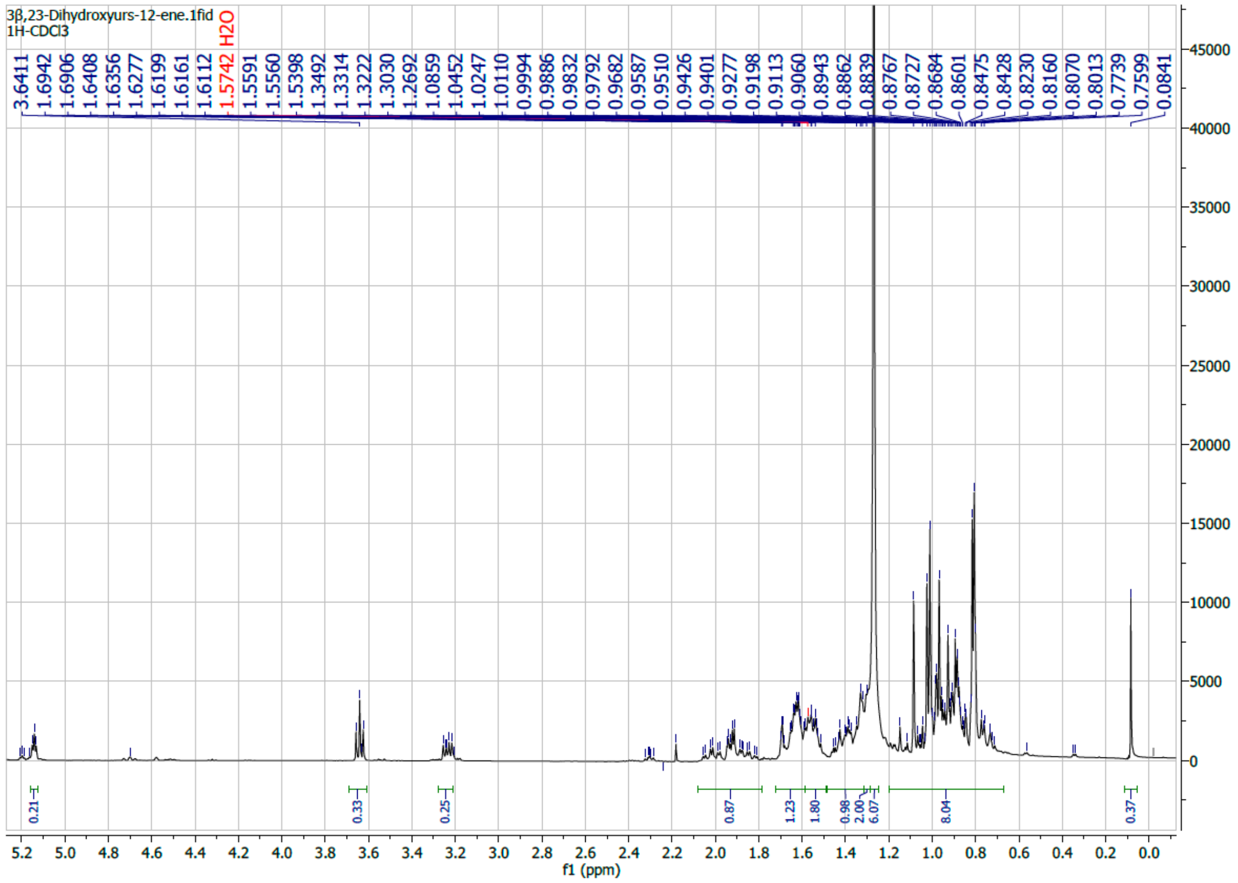

Figure S1. ^{1}H NMR spectrum of 3β,23-Dihydroxyurs-12-ene (**1**)

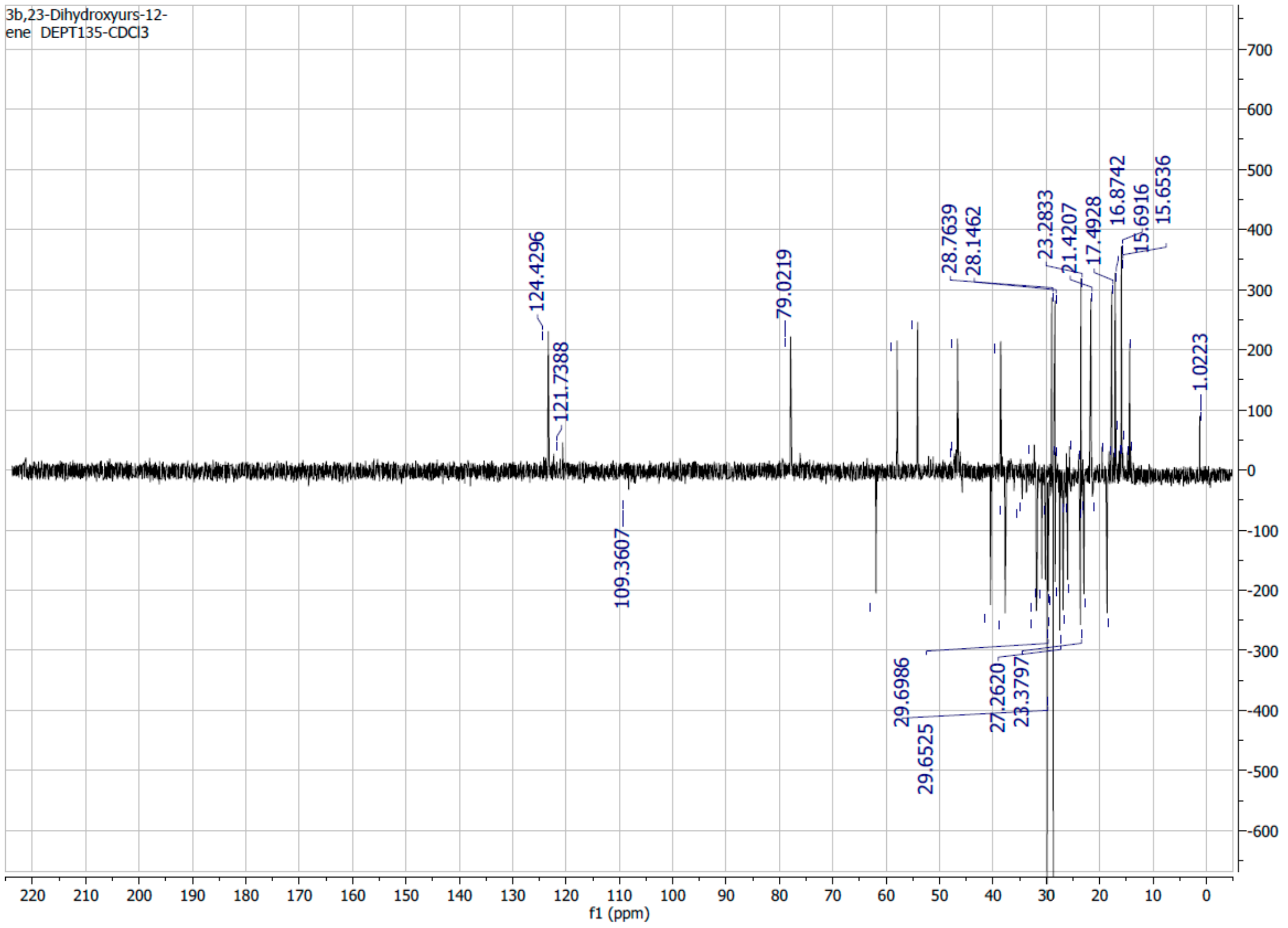

Figure S2. DEPT135 NMR spectrum of 3β,23-Dihydroxyurs-12-ene (**1**)

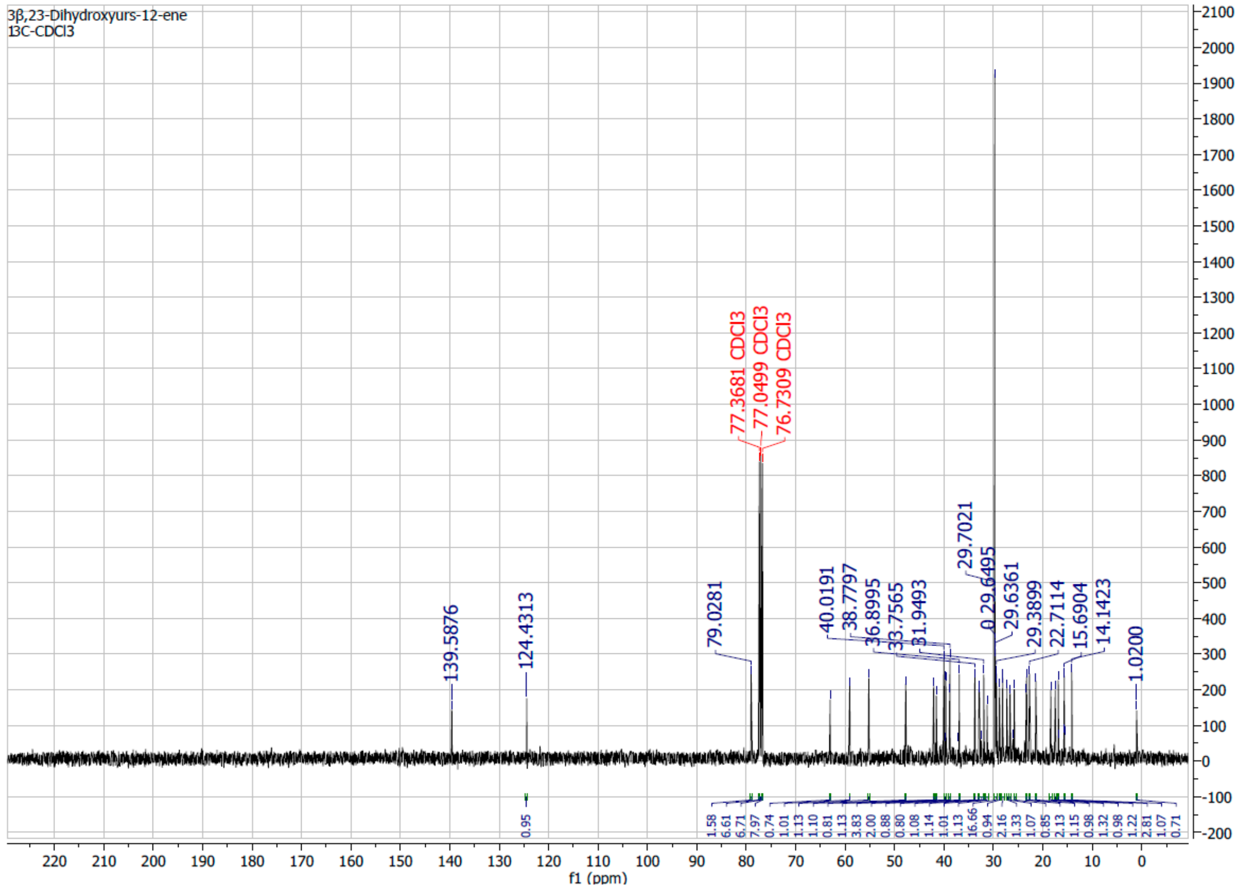

Figure S3. ^{13}C NMR spectrum of 3β,23-Dihydroxyurs-12-ene (**1**)

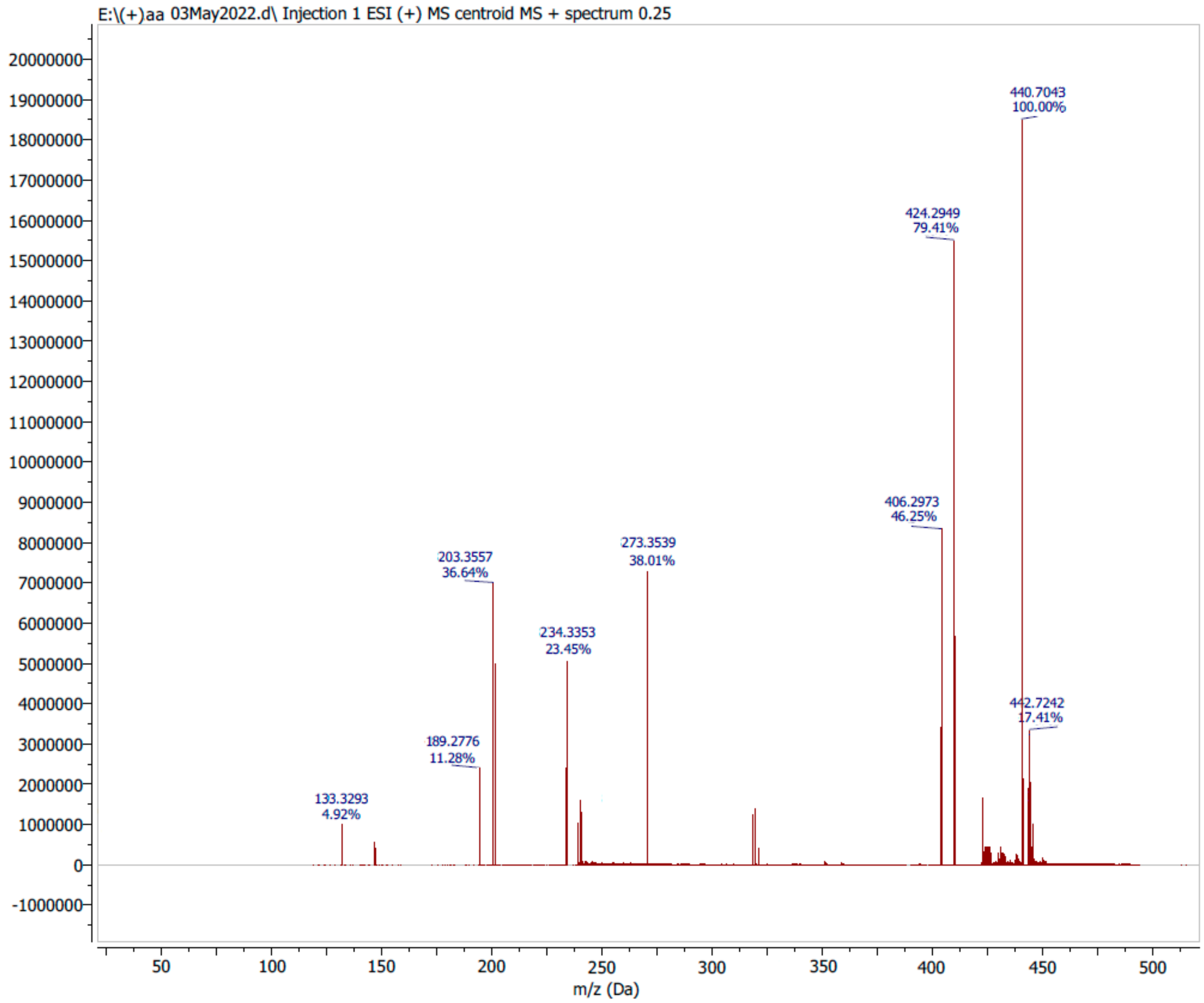

Figure S4. Mass spectrum of 3β,23-Dihydroxyurs-12-ene (**1**)

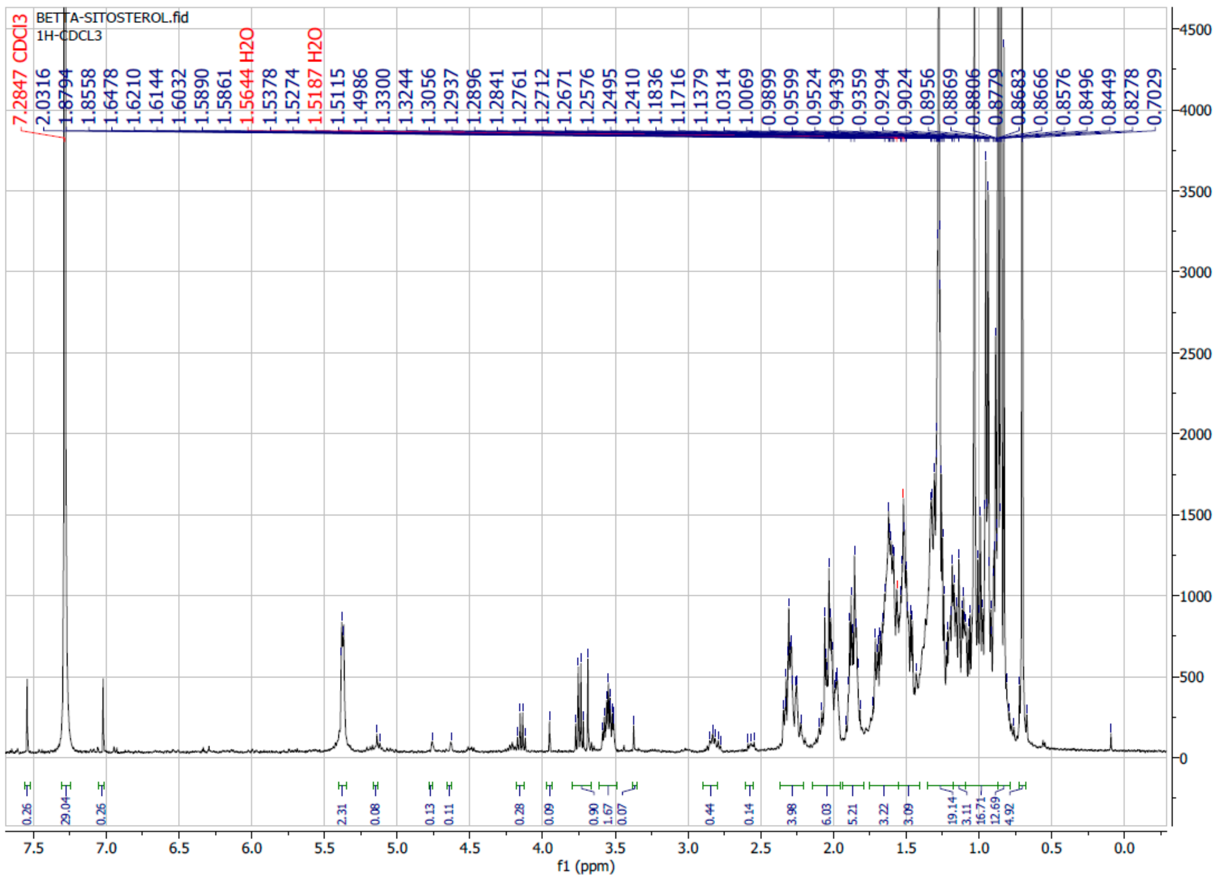

Figure S5. ^{1}H NMR spectrum of β-sitosterol (**2**)

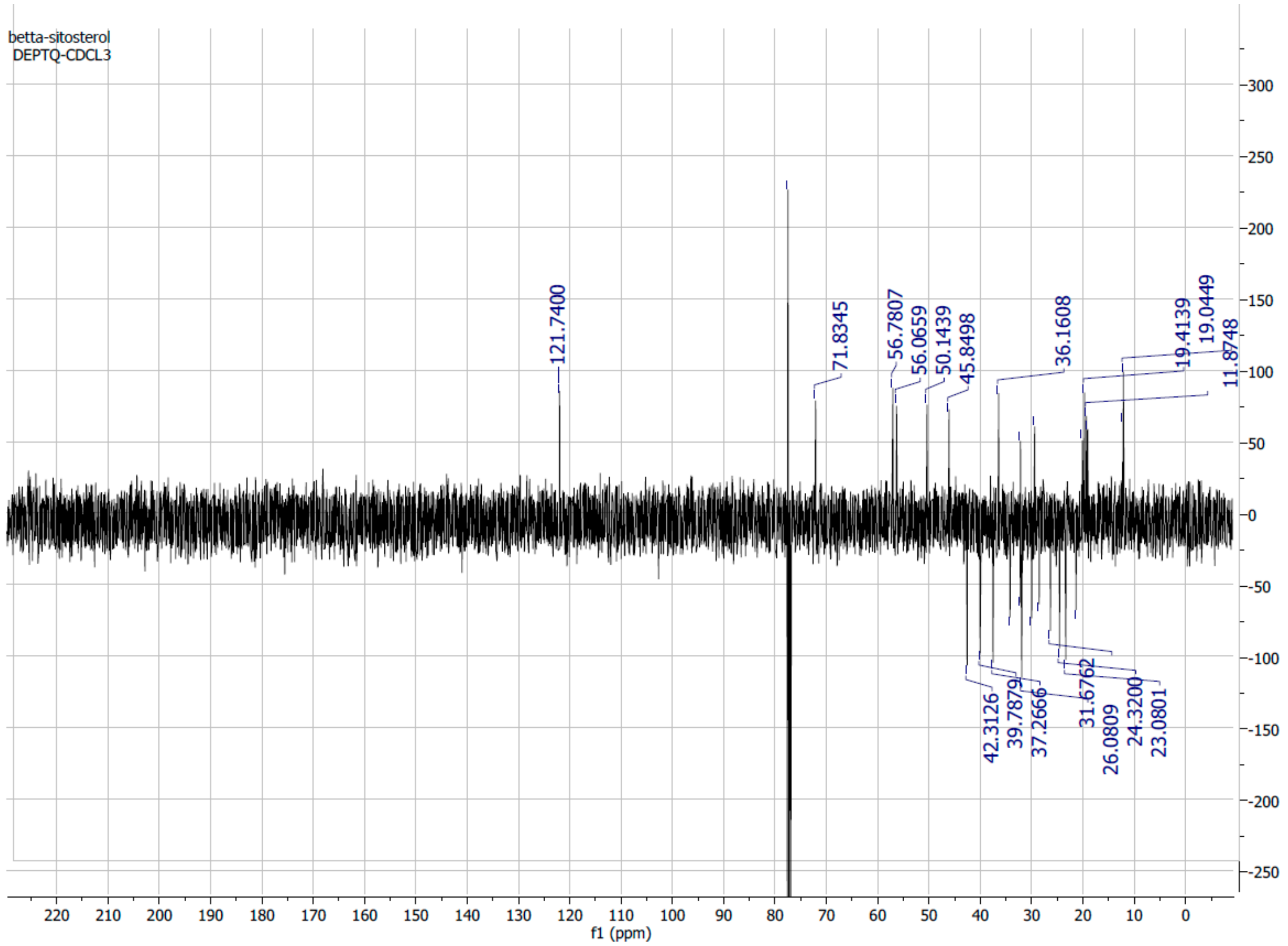

Figure S6. DEPTQ NMR spectrum of β-sitosterol (**2**)

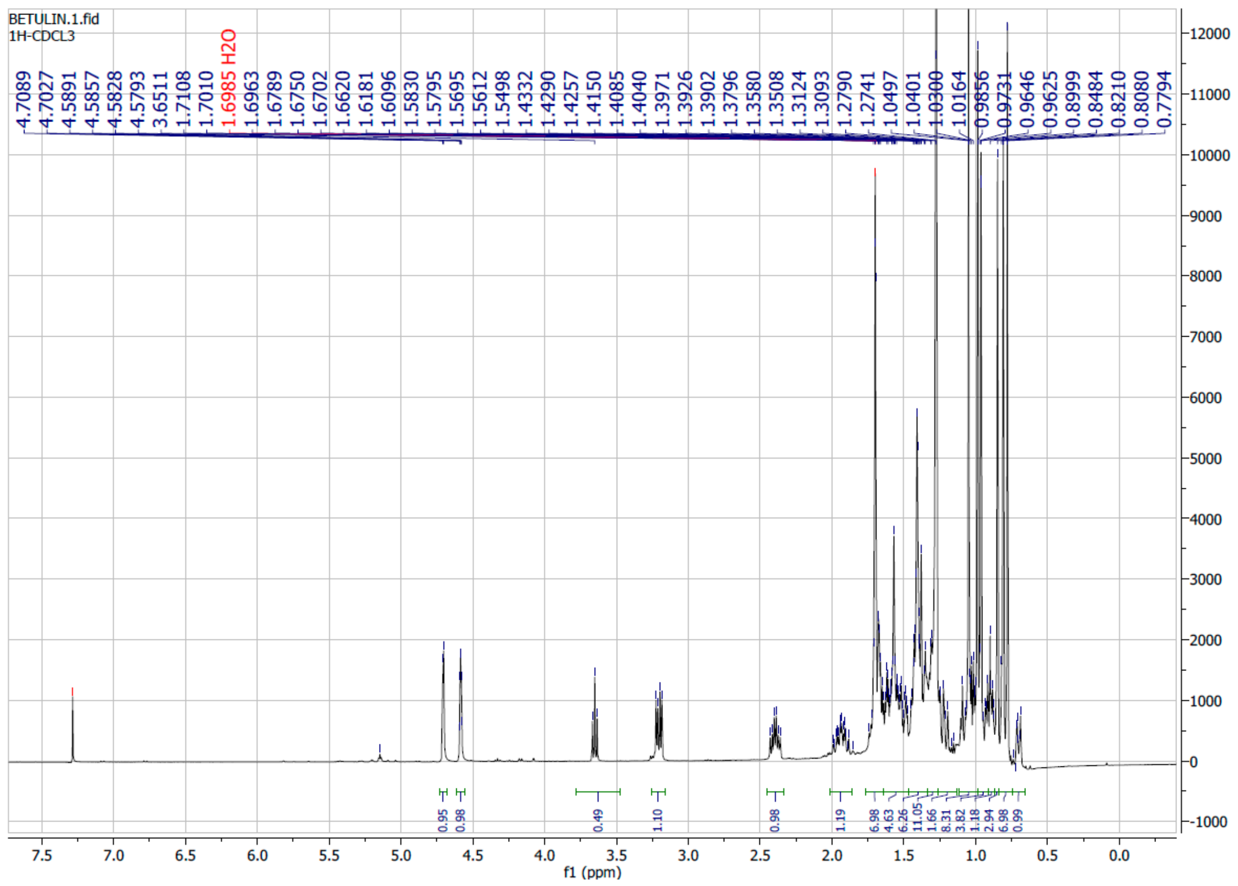

Figure S7. ^{1}H NMR spectrum of Betulin (**3**)

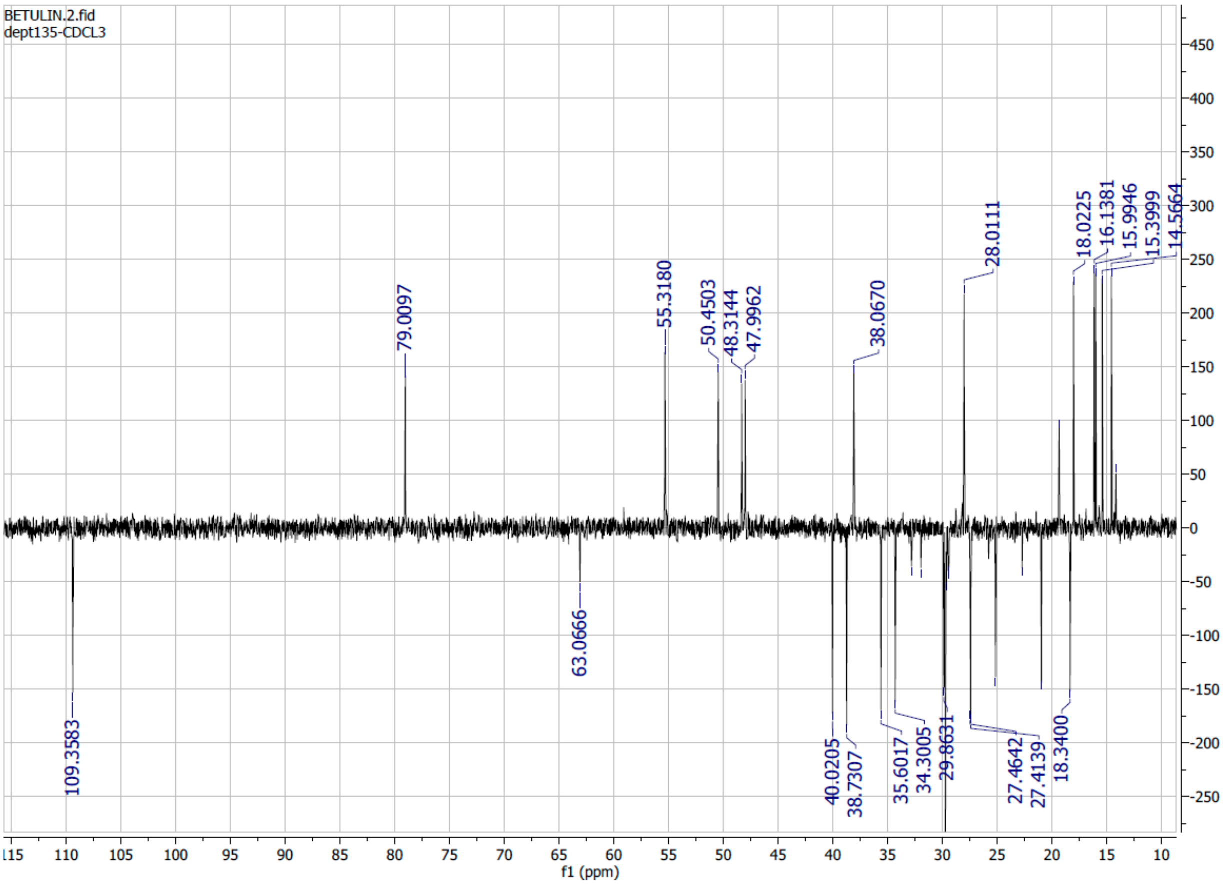

Figure S8. DEPT135 NMR spectrum of Betulin (**3**)

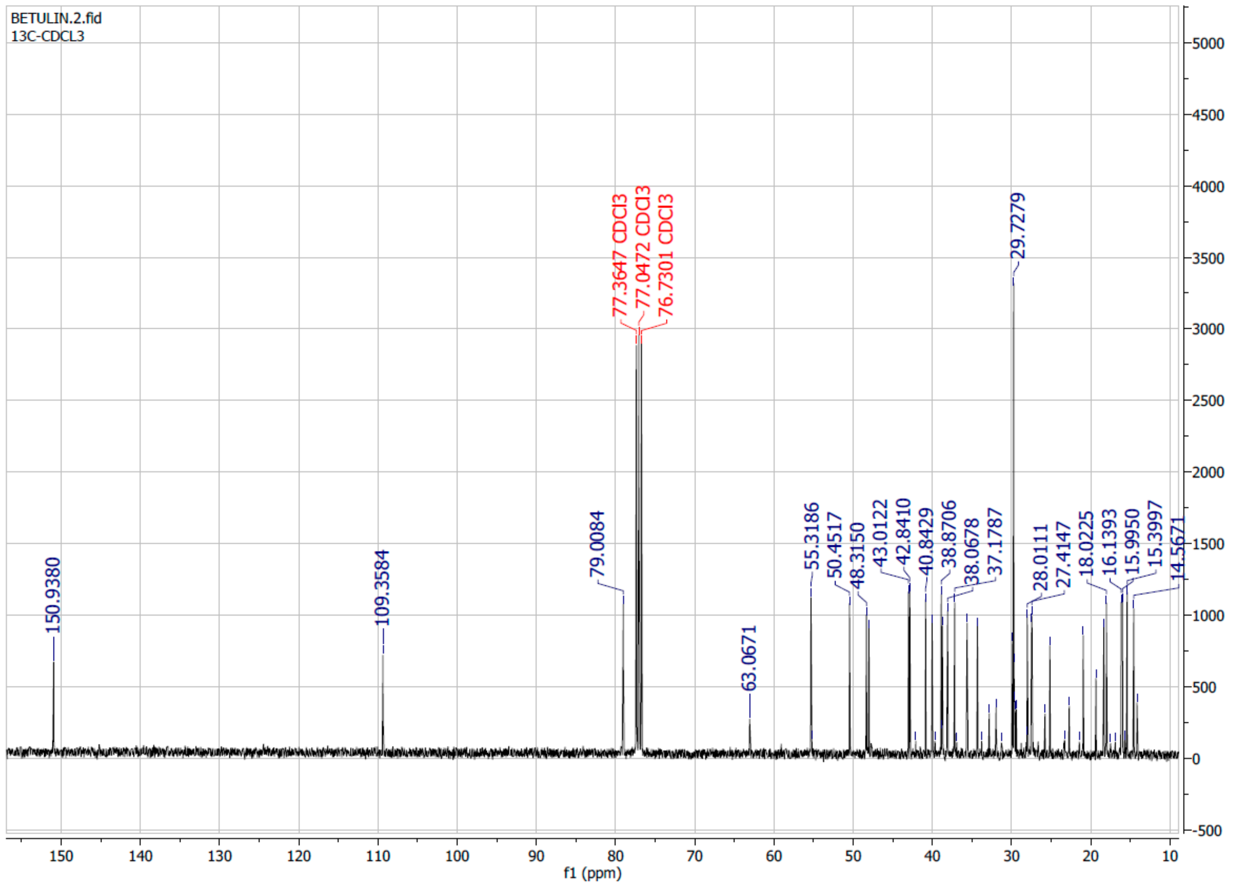

Figure S9. ^{13}C NMR spectrum of Betulin (**3**)

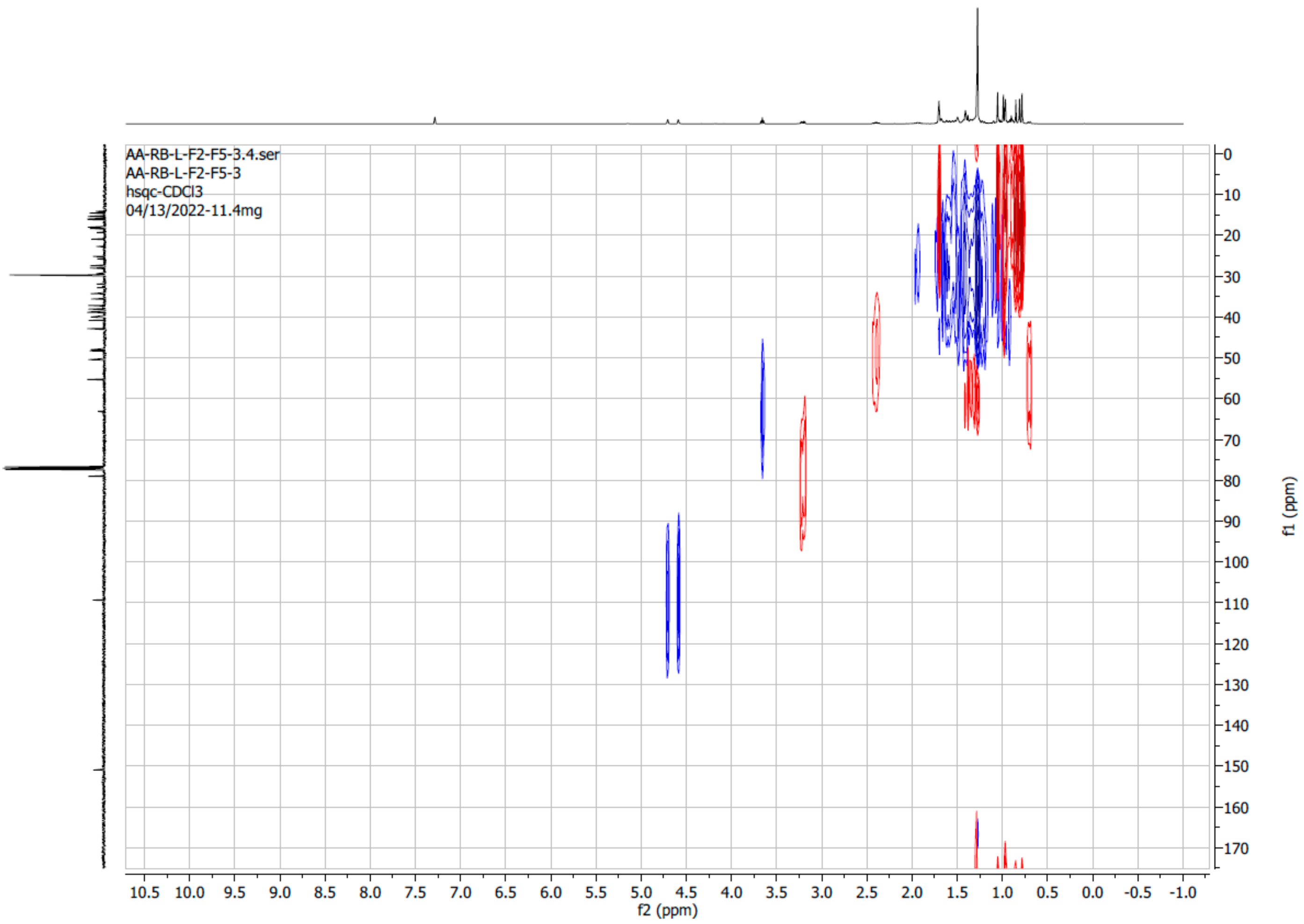

Figure S10. HSQC NMR spectrum of Betulin (**3**)

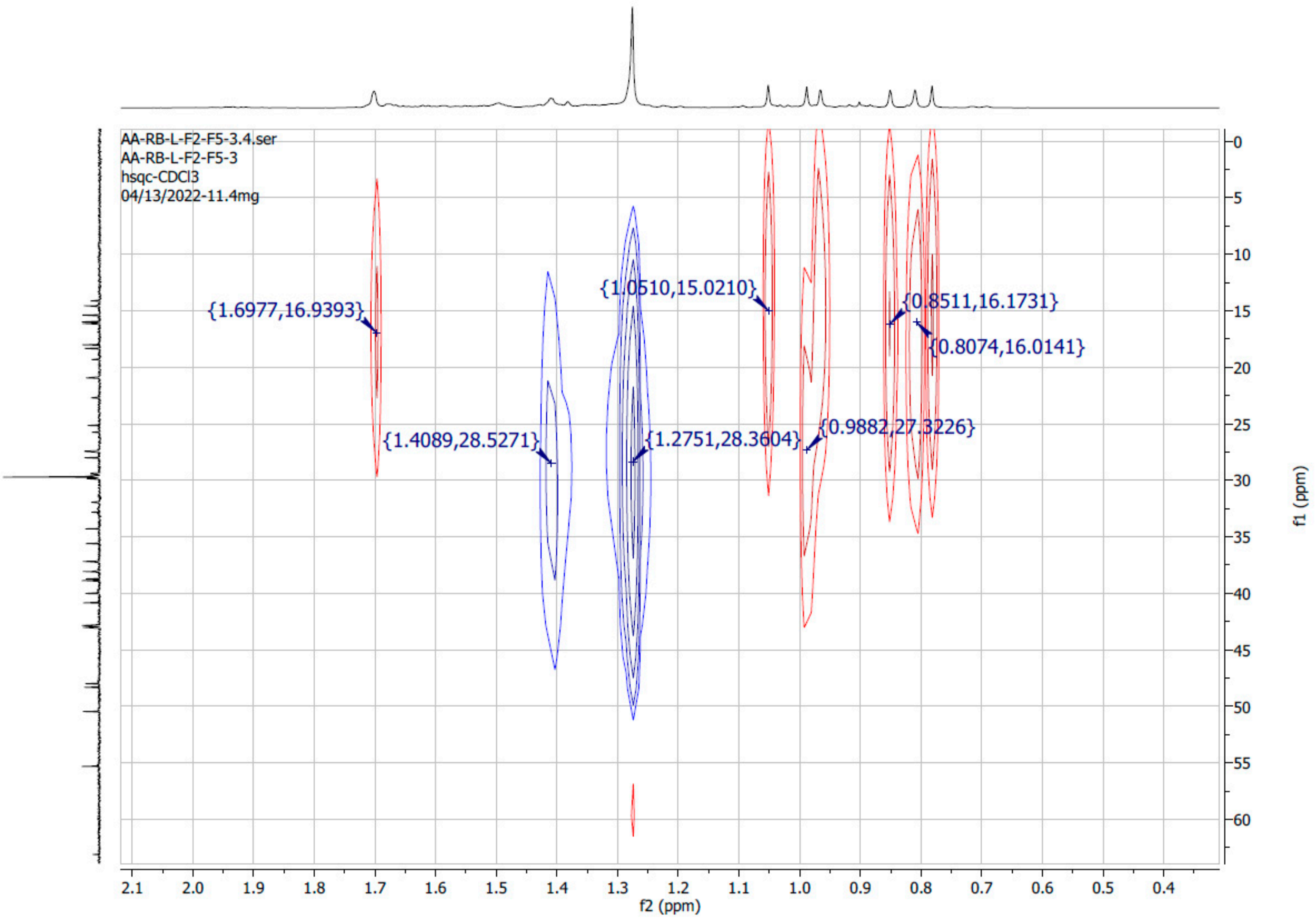

Figure S11. HSQC NMR spectrum of Betulin (**3**)

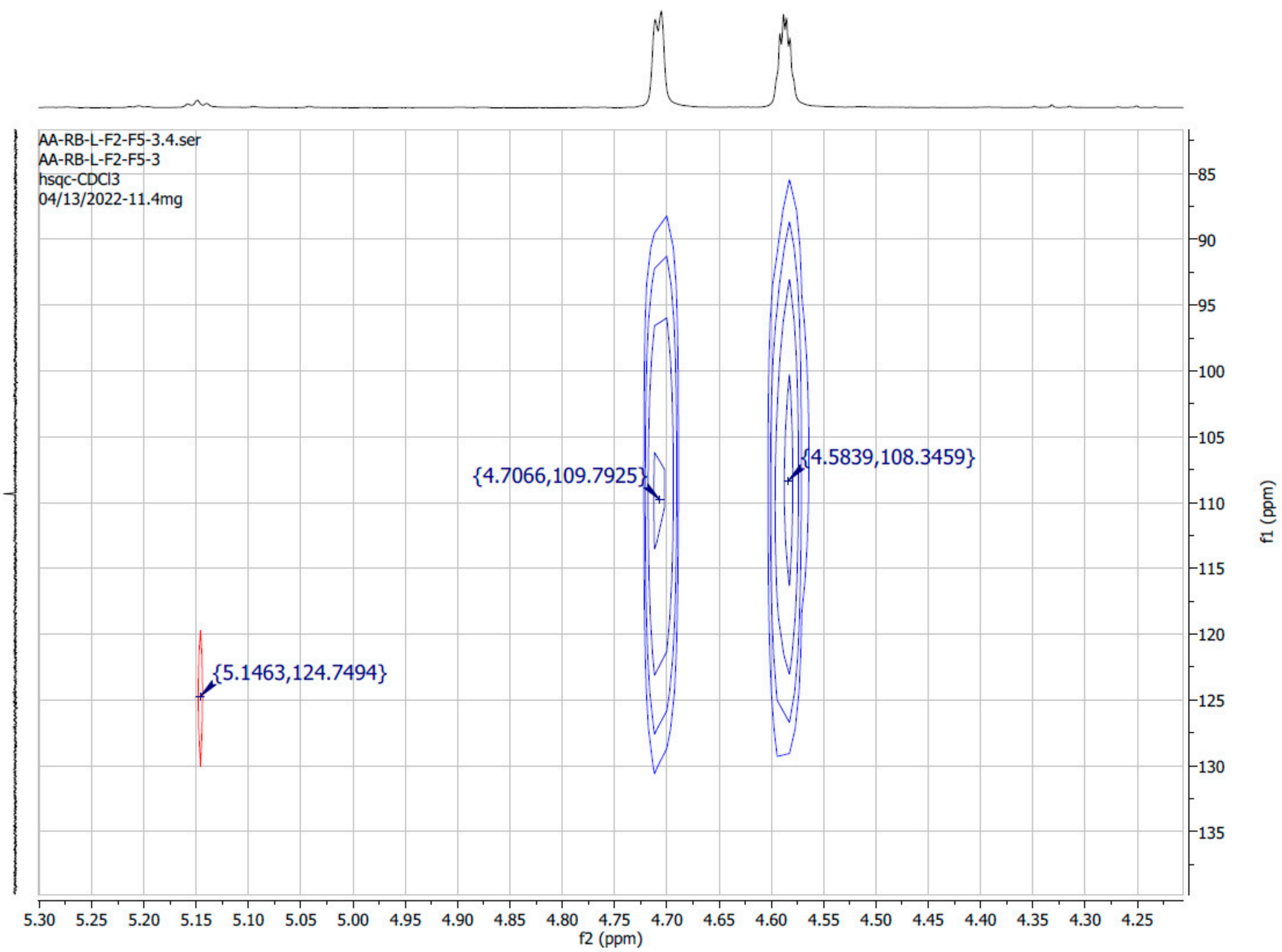

Figure S12. HSQC NMR spectrum of Betulin (**3**)

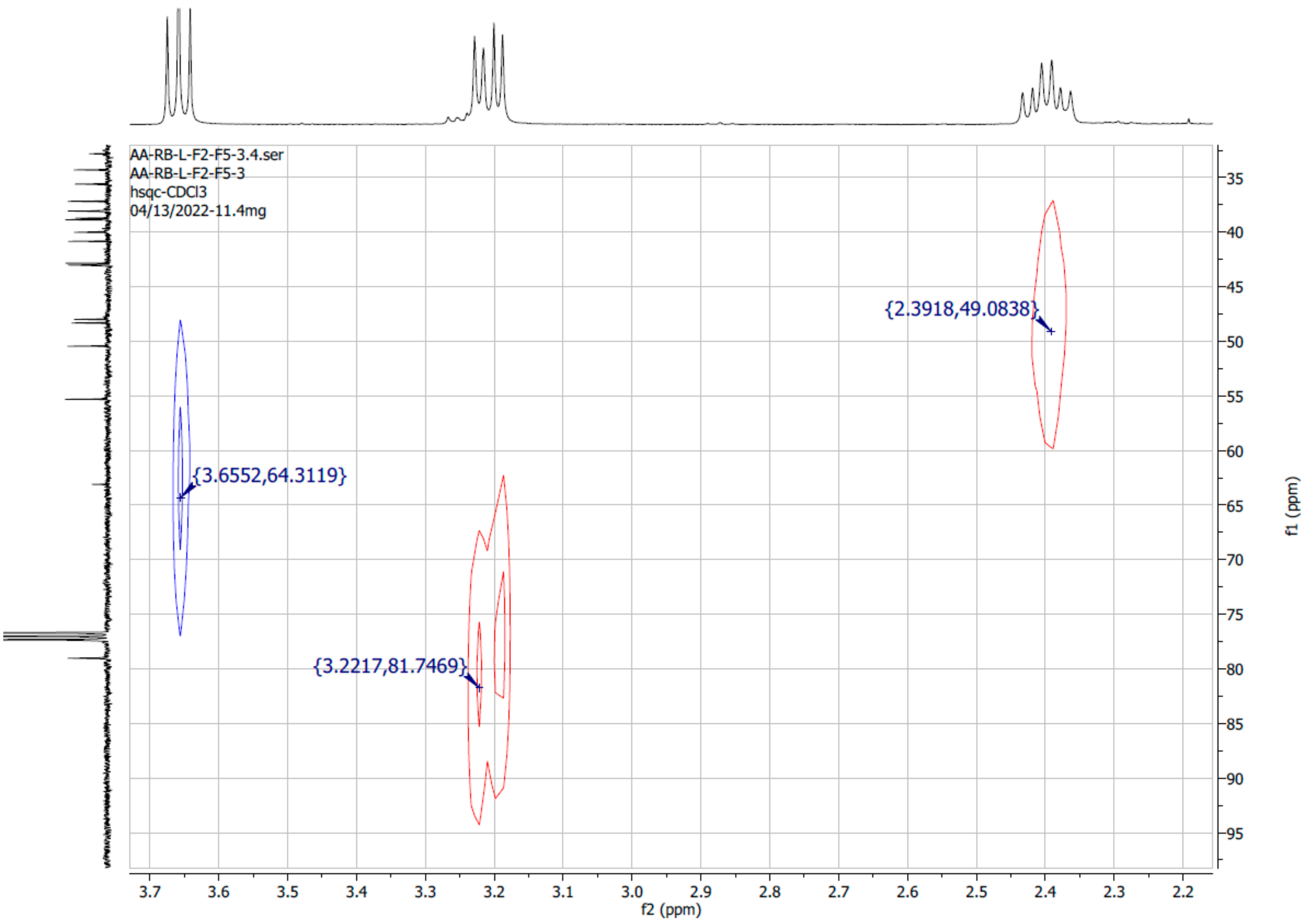

Figure S13. HSQC NMR spectrum of Betulin (**3**)

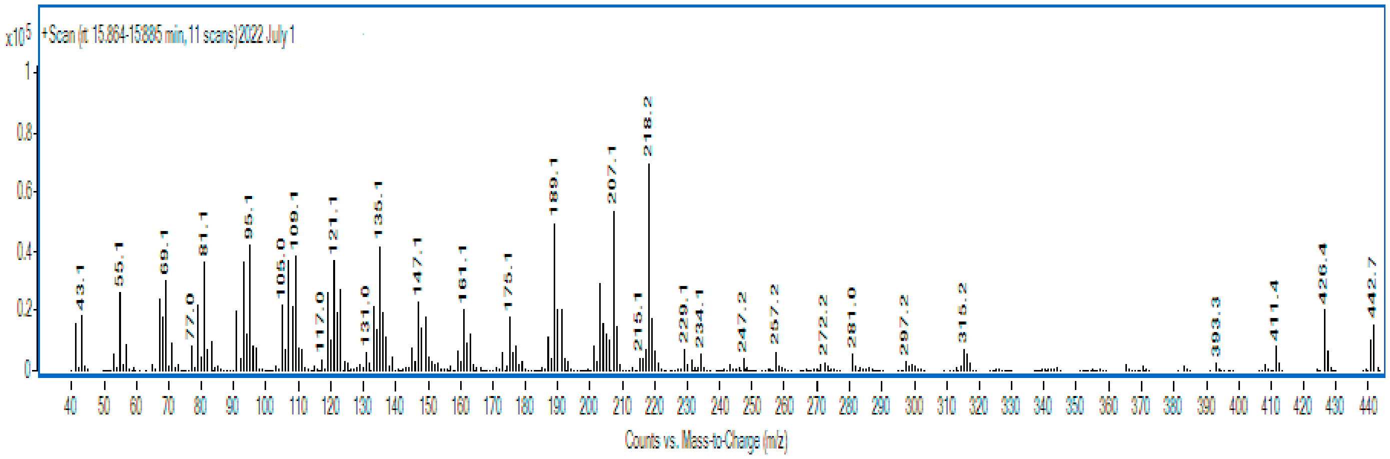

Figure S14. GC-MS spectrum of Betulin (**3**)

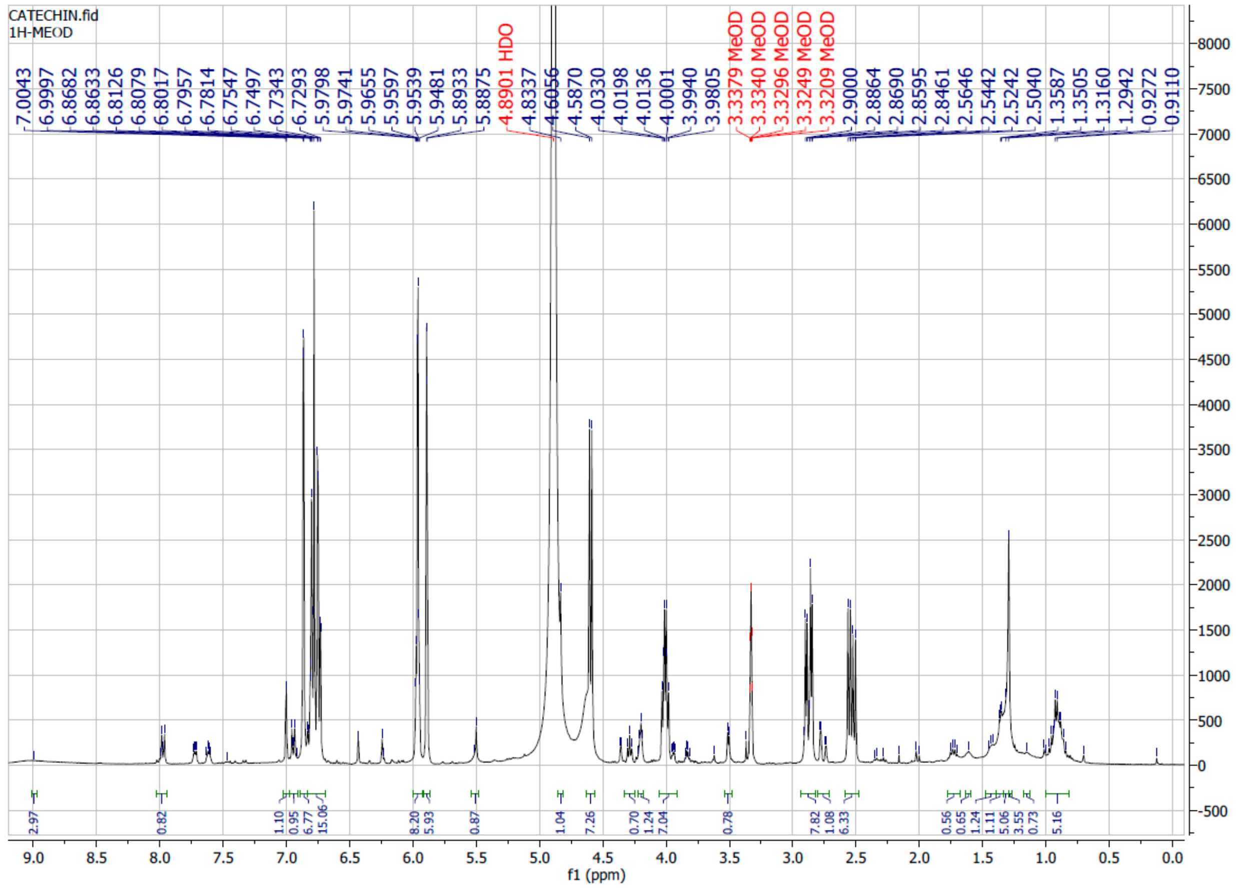

Figure S15. ^{1}H NMR spectrum of (+)-Catechin (4)

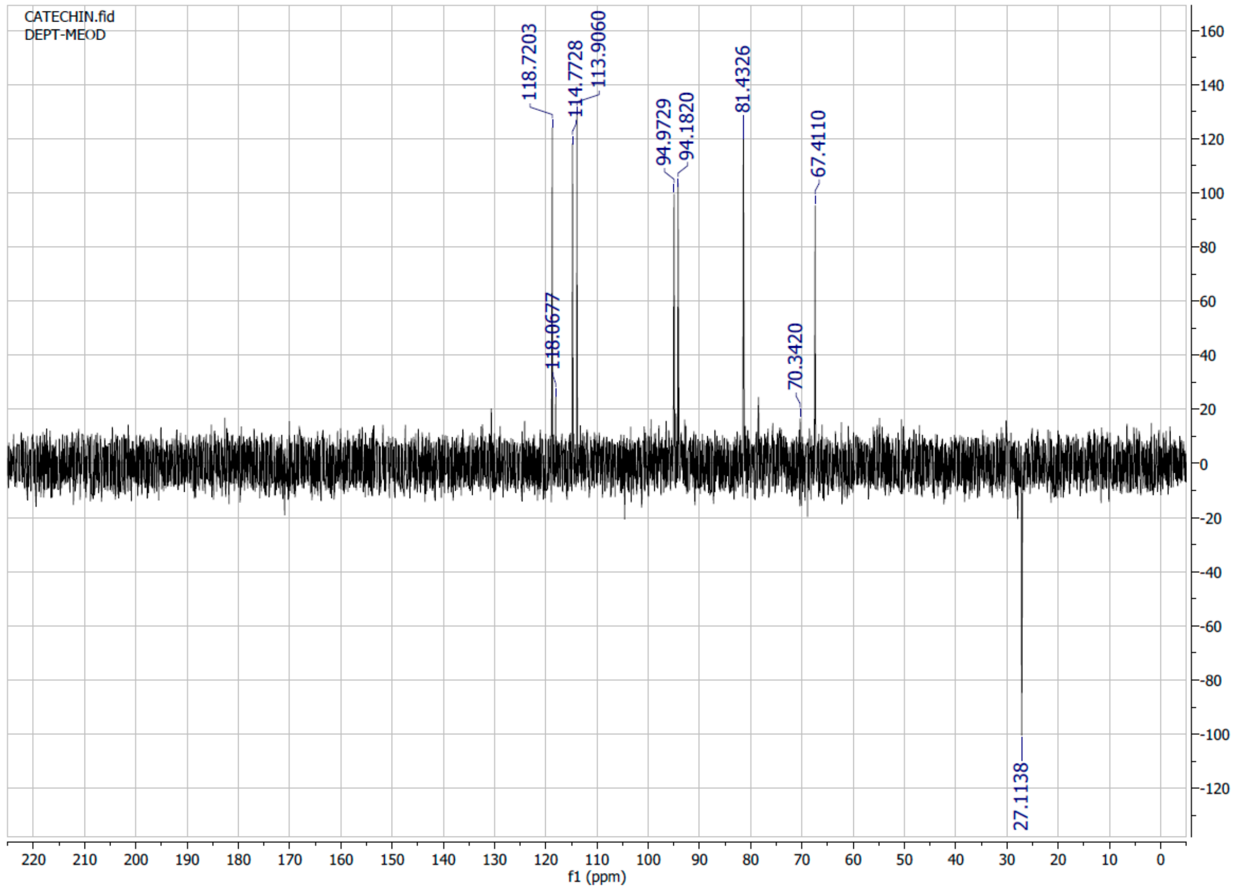

Figure S16. DEPT NMR spectrum (+)-Catechin (4)

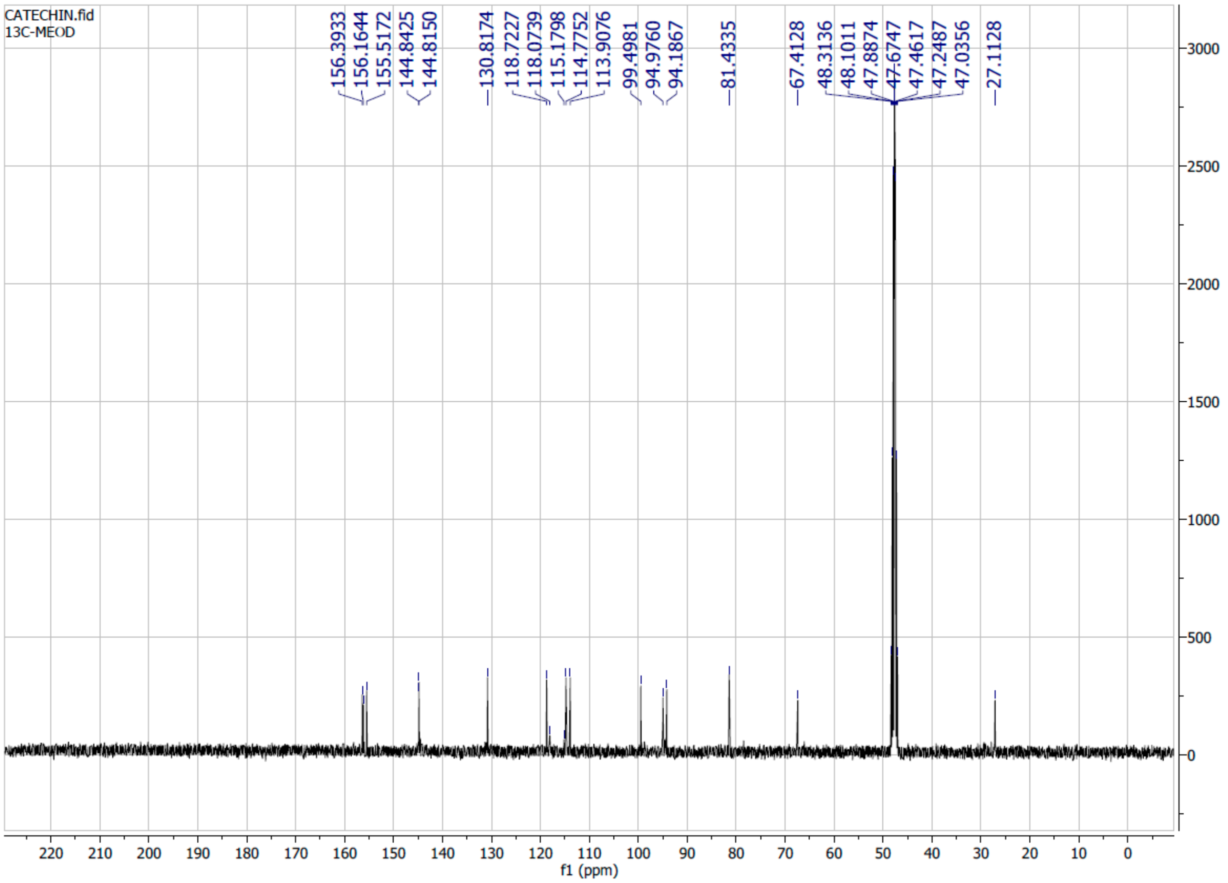

Figure S17. ^{13}C NMR spectrum of (+)-Catechin (4)

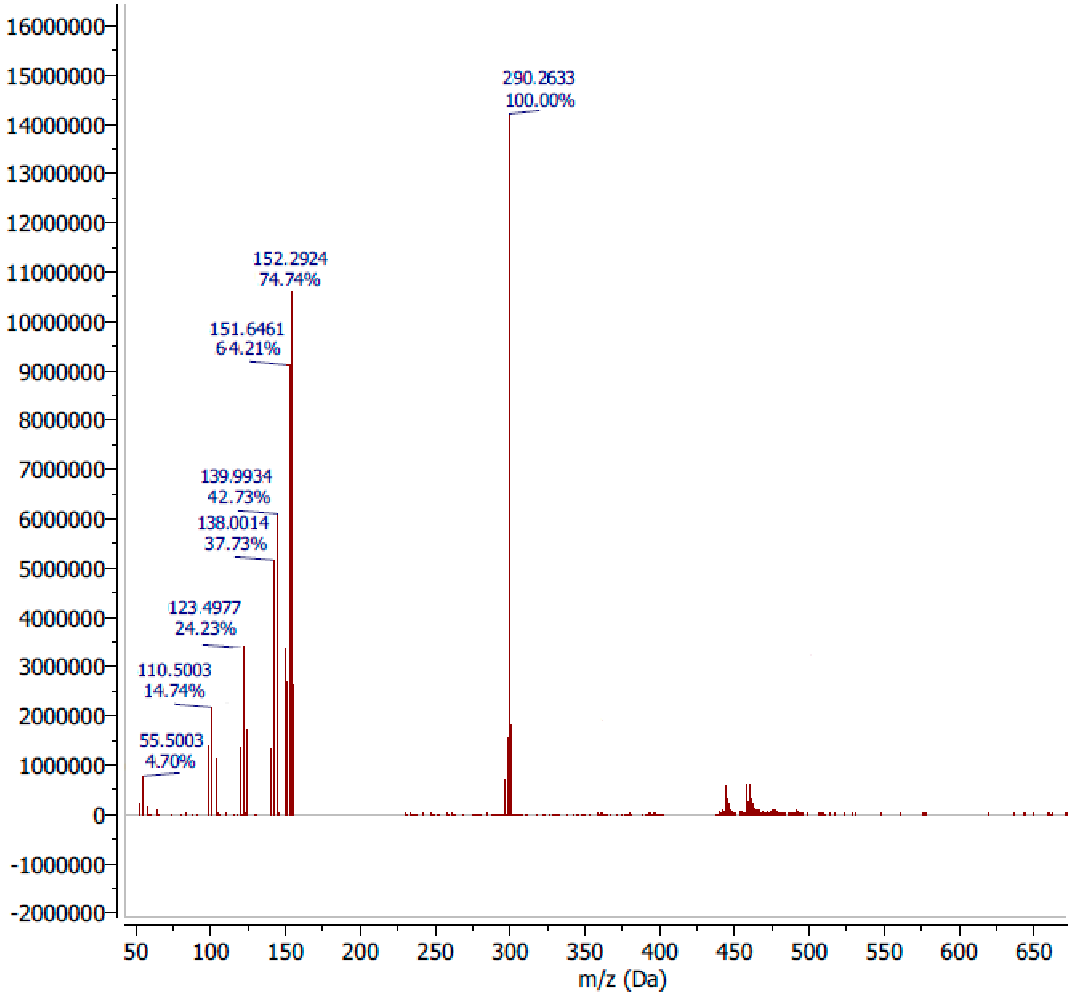

Figure S18. Mass spectrum of (+)-Catechin (4)

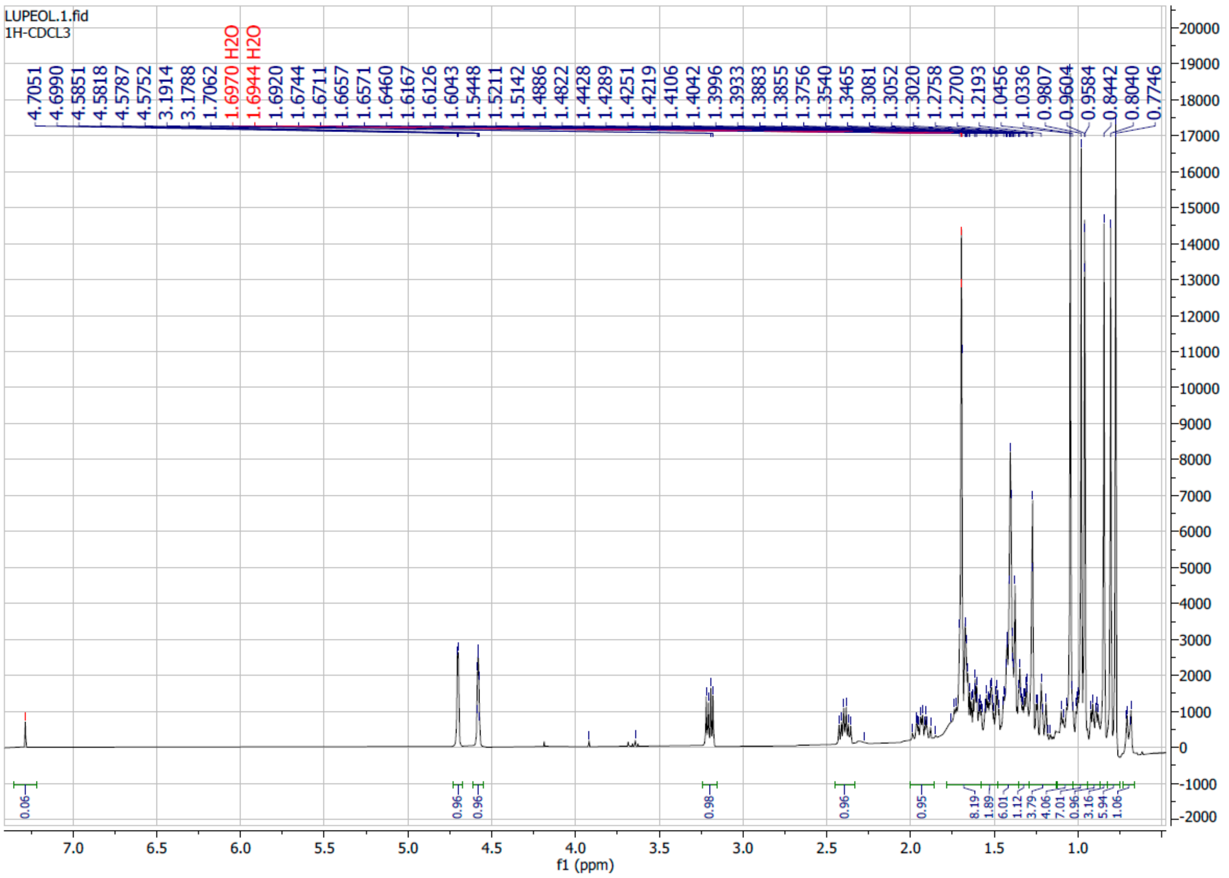

Figure S19. ^1H NMR spectrum of Lupeol (5)

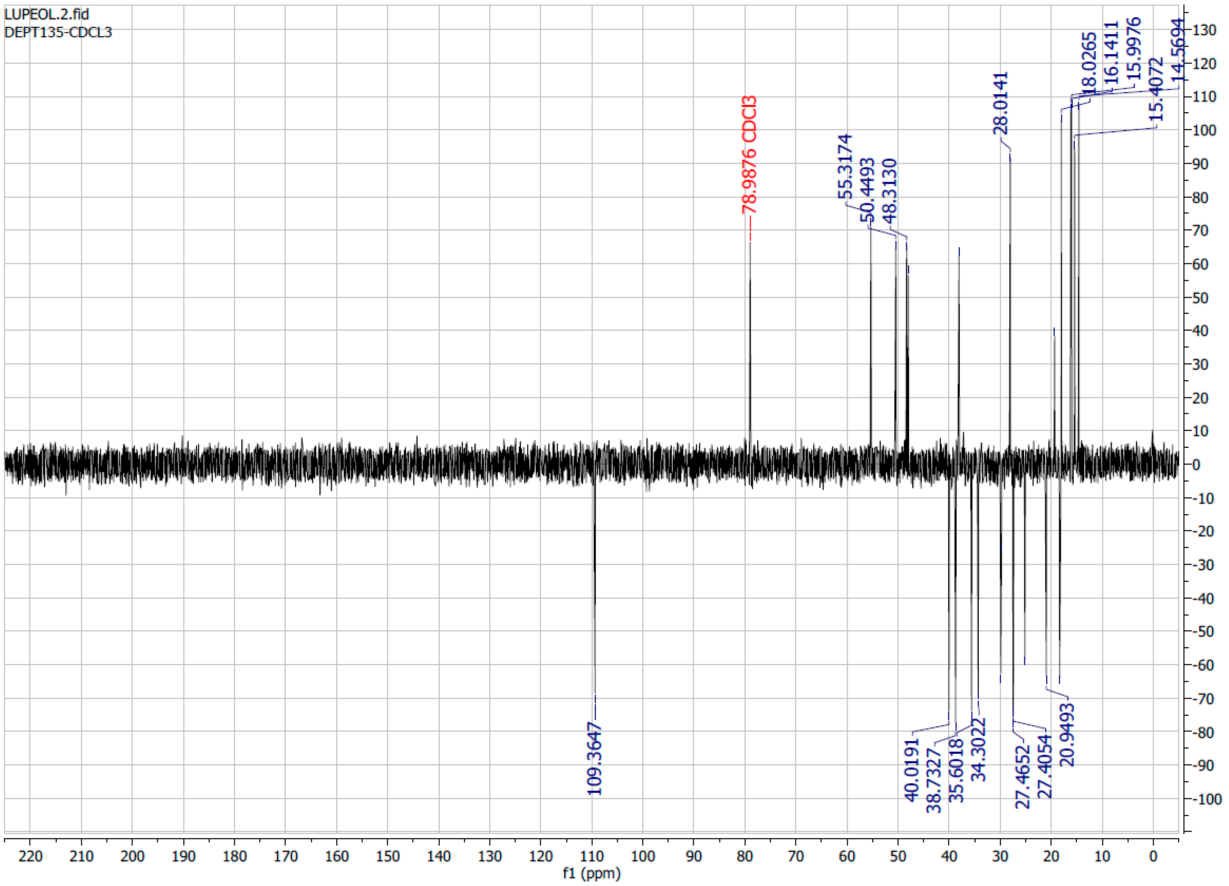

Figure S20. DEPT135 NMR spectrum of Lupeol (5)

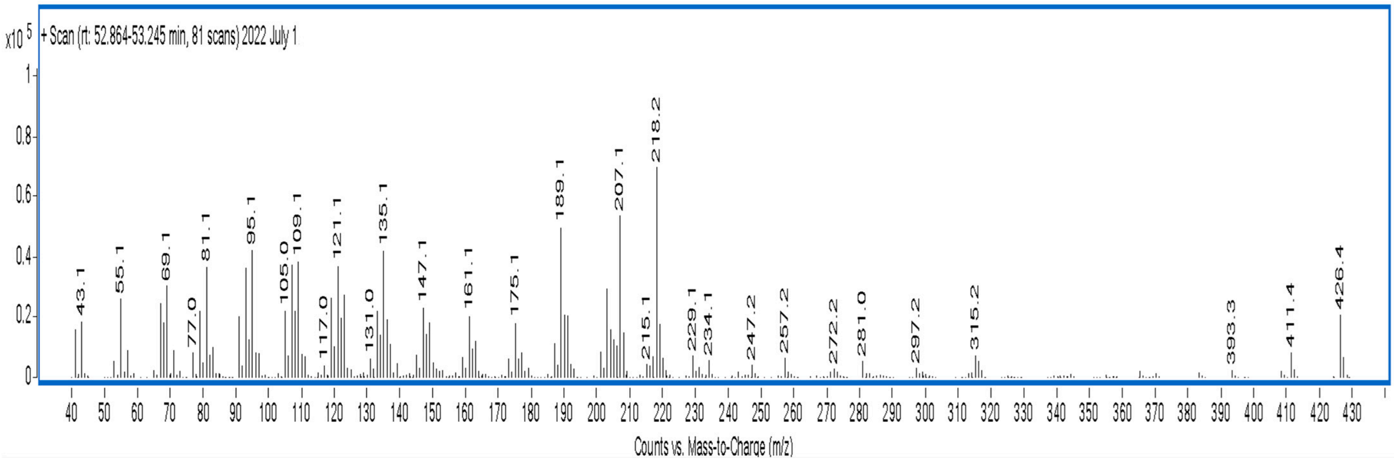

Figure S21. GC-MS Data of Lupeol (5)

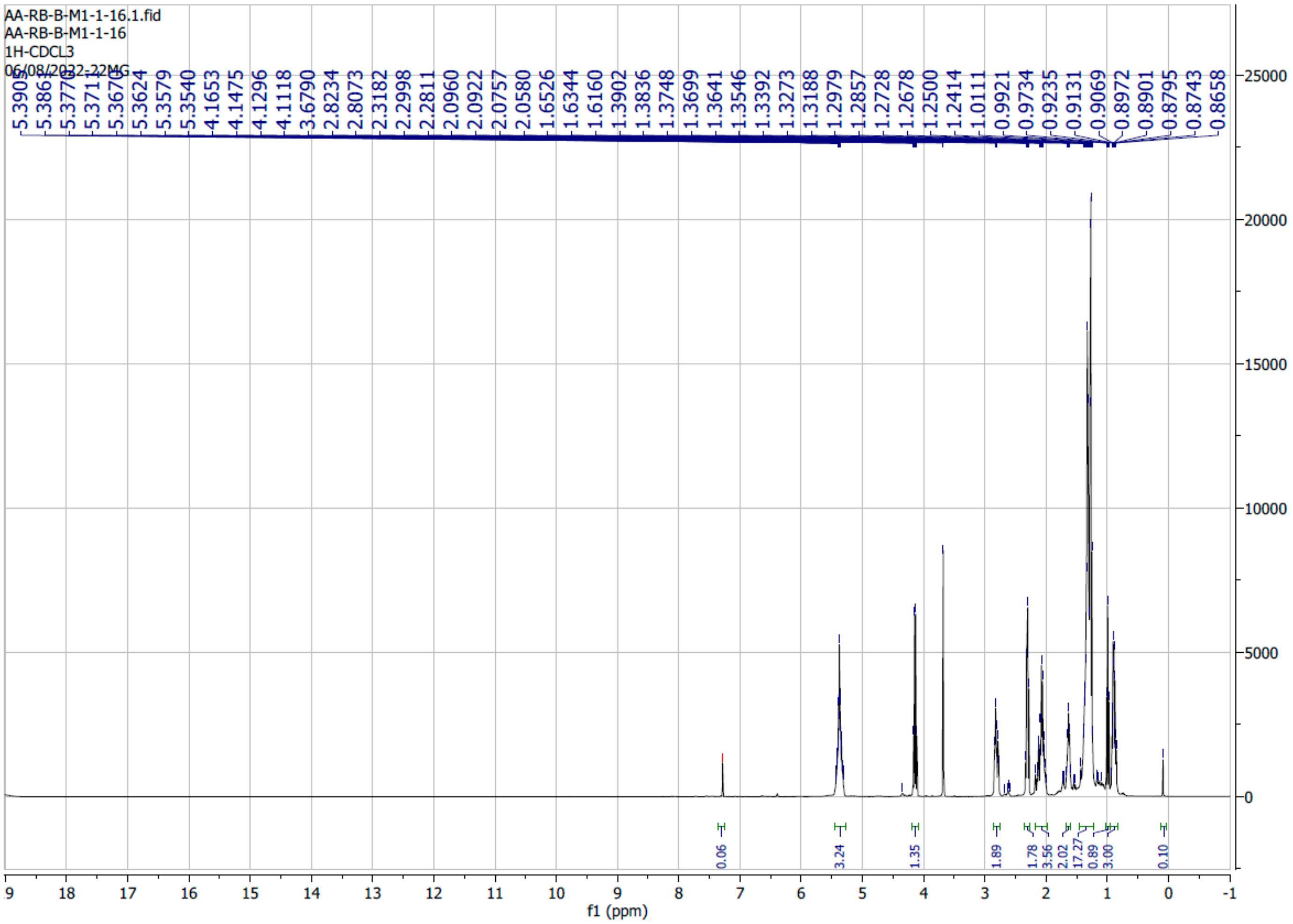

Figure S22. ^{1}H NMR spectrum of Ehyl linoleate (6) and Ethyl linolenoate (7)

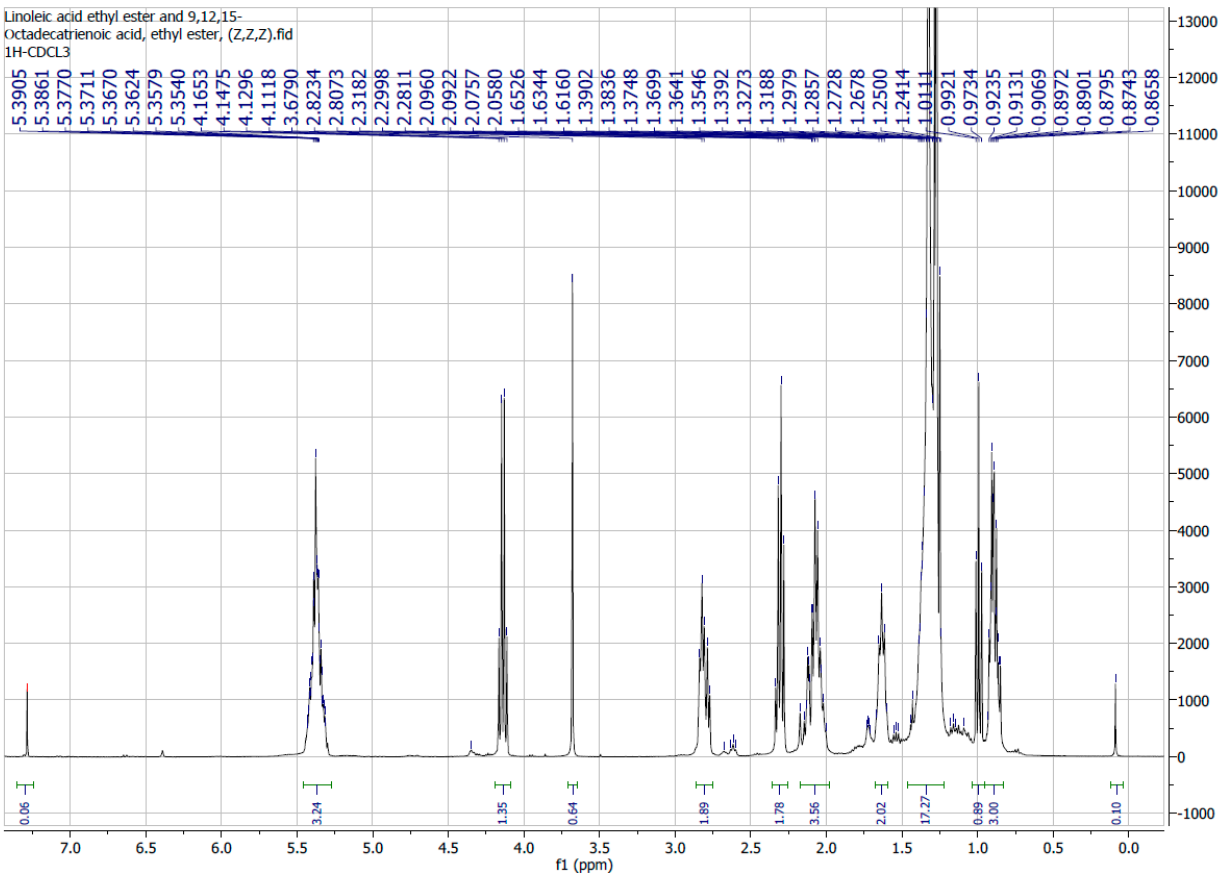

Figure S23. ^{1}H NMR spectrum of Ehyl linoleate (6) and Ethyl linolenoate (7)

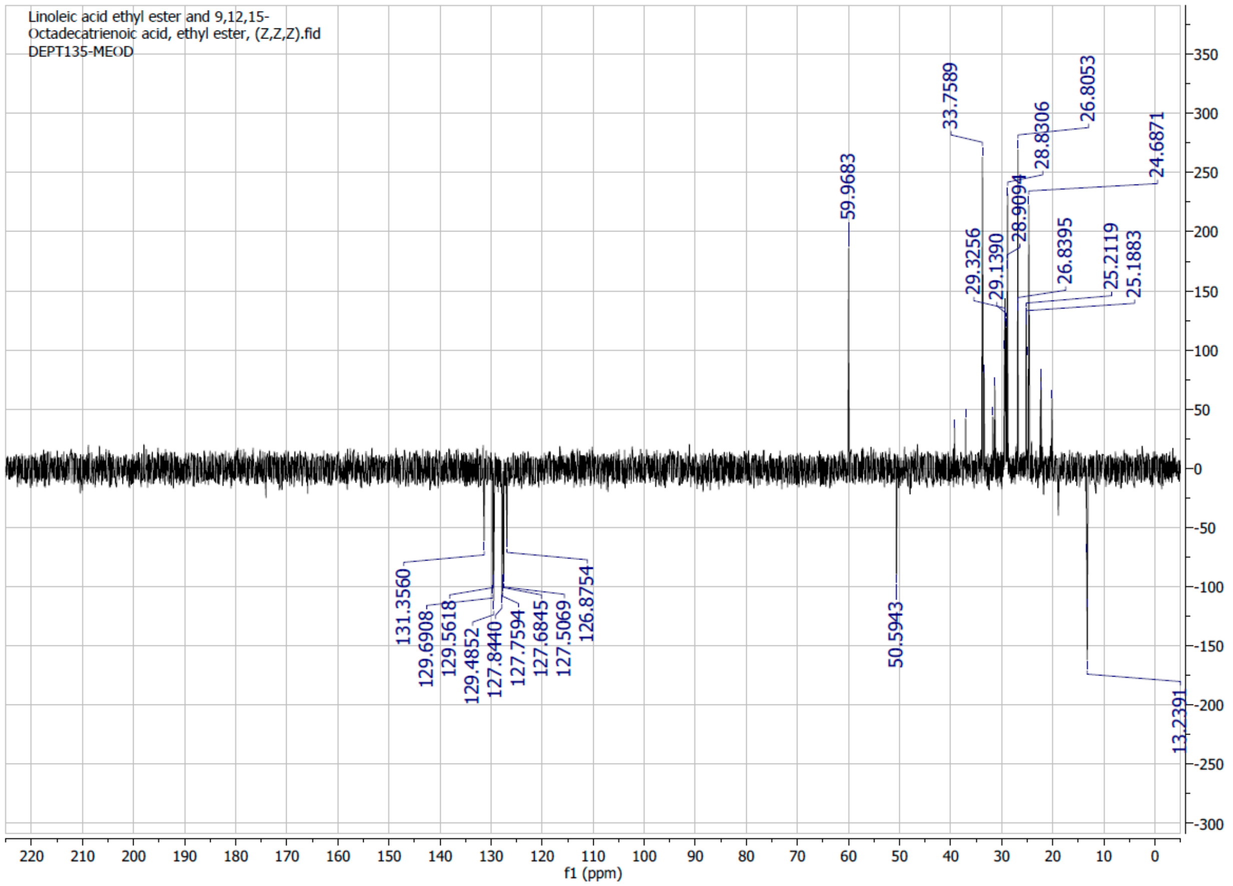

Figure S24. DEPT135 NMR spectrum of Ehyl linoleate (6) and Ethyl linolenoate (7)

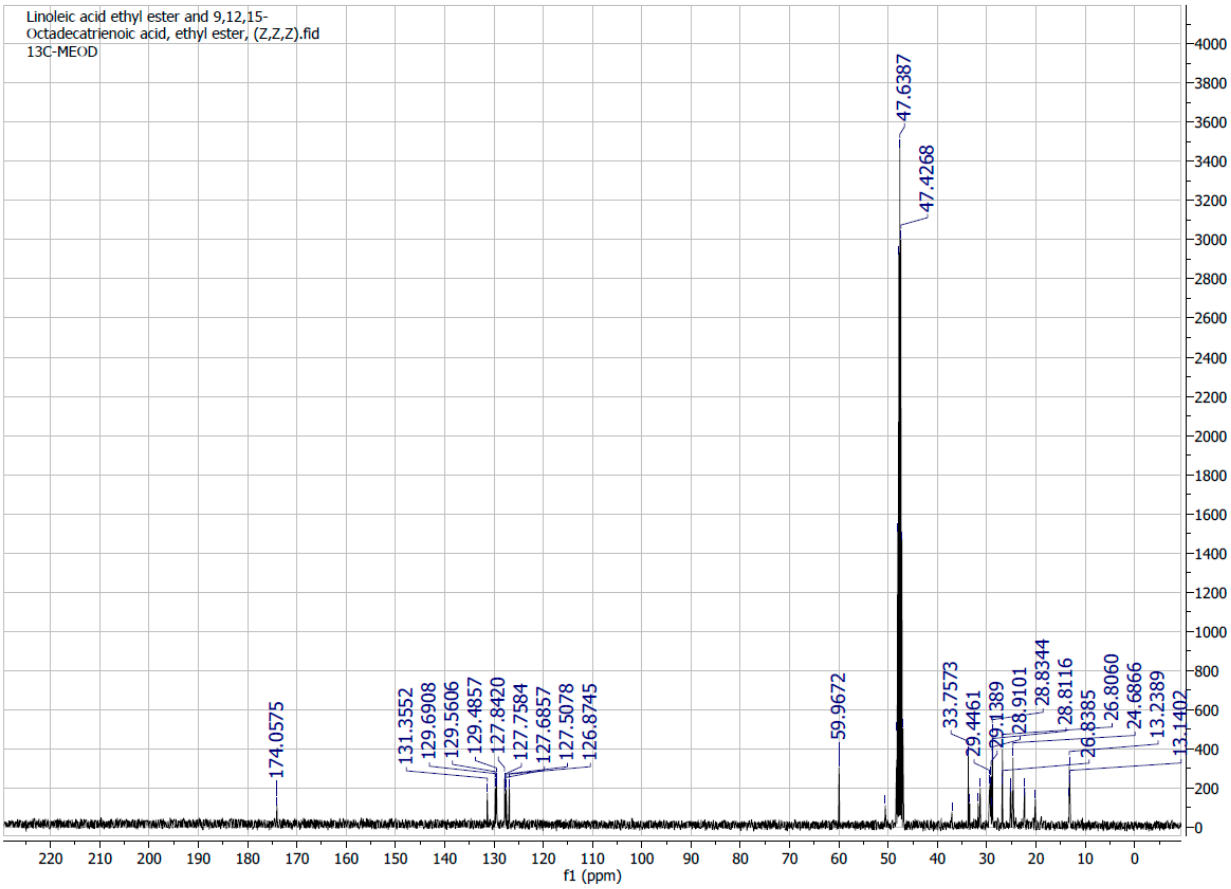

Figure S25. ^{13}C NMR spectrum of Ehyl linoleate (6) and Ethyl linolenoate (7)

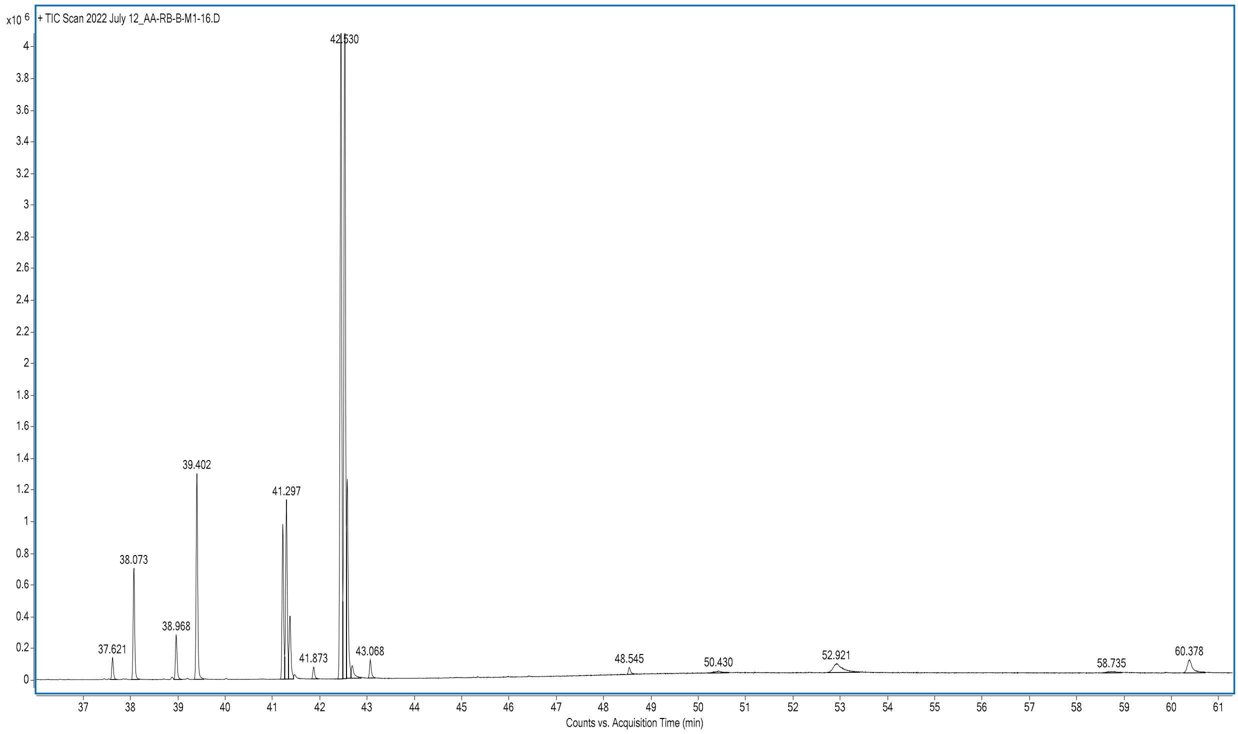

Figure S26. GC-MS Data of Ethyl linoleate (6) and Ethyl linolenoate (7)

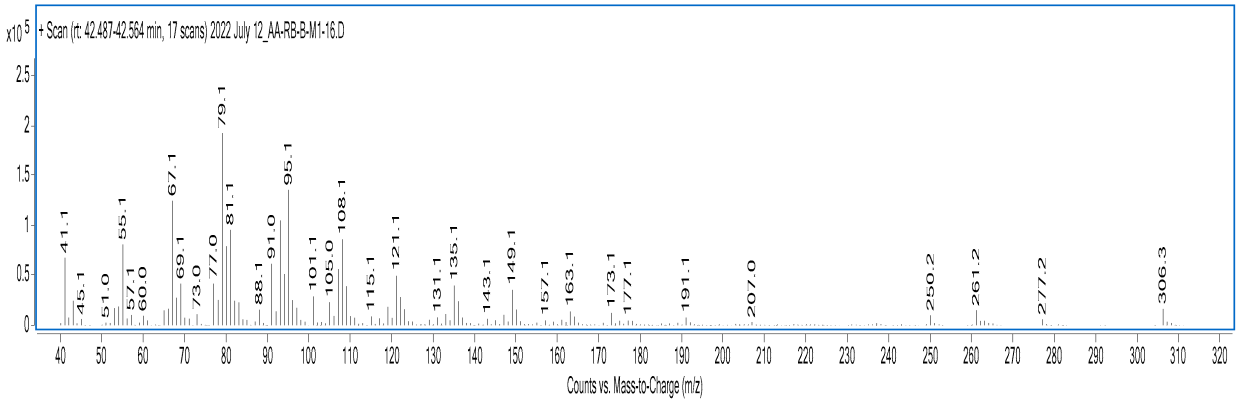

Figure S27. GC-MS Data of Ethyl linolenoate

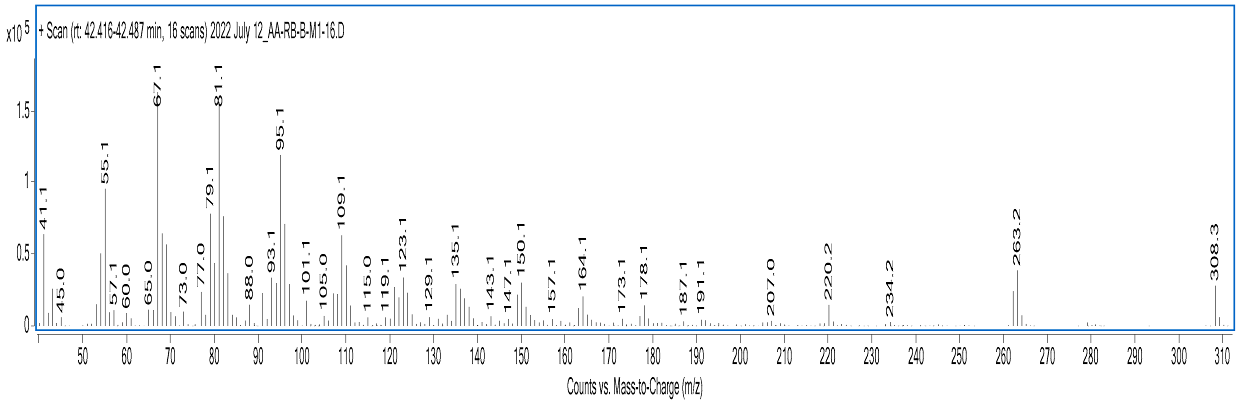

Figure S28. GC-MS Data of Ethyl linoleate
